# Use of Luminescence Modulation in a New Series of Mixed Lanthanide Metal–Organic Frameworks for Selective Firearm Ammunition Marking

**DOI:** 10.1021/acsomega.4c08401

**Published:** 2024-12-12

**Authors:** Júlia P. De Oliveira Silva, Marcos V. Colaço, Alexandre de Resende Camara, Renato de Almeida Pereira, Eduardo de Oliveira Fernandes, Claudiane Costa Canuto, Diego Rissi Carvalhosa, Lippy Faria Marques

**Affiliations:** † Grupo de Química de Coordenação e Espectroscopia de Lantanídeos (GQCEL), Instituto de Química, Universidade do Estado do Rio de Janeiro, Rio de Janeiro, RJ 20550-013, Brazil; ‡ Laboratório de Física Médica (LabFisMed), Instituto de Física, Universidade do Estado do Rio de Janeiro, Rio de Janeiro, RJ 25550-013, Brazil; § Departamento de Eletrônica Quântica, Instituto de Física, Universidade do Estado do Rio de Janeiro, Rio de Janeiro, RJ 25550-013, Brazil

## Abstract

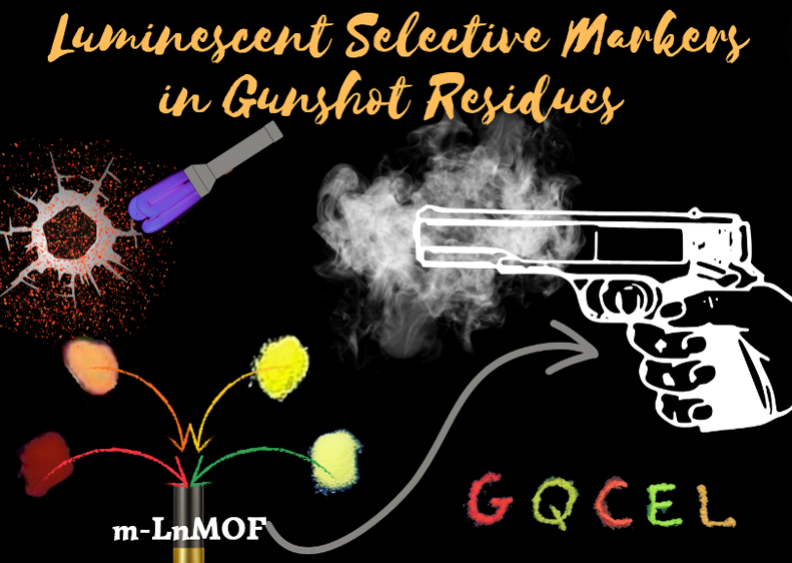

Metal–organic frameworks (MOFs) are coordination polymers that can generally be described by secondary building units (SBUs). These include lanthanide MOFs, specifically mixed lanthanide MOFs (m-LnMOFs), encompassing coordination polymers formed by two or more different Ln^3+^ ions. These compounds have been the subject of study by inorganic chemists worldwide, mainly due to the possibility of obtaining long-awaited tunable luminescence, i.e., compounds that emit luminescence in various visible spectrum regions. A wide range of emission color scan be obtained by inserting different Eu^3+^, Tb^3+^, and Gd^3+^ ion molar fractions in m-LnMOFs, which can also be adjusted by choosing the Ln^3+^ ion or its amount in each network. This study presents a new series of m-LnMOFs supported by 1,2,4,5-benzenetetracarboxylic acid (H_4_btec). These compounds were completely characterized in their solid state through different analytical and spectroscopic techniques, and an in-depth photophysical study was conducted. Due to their high thermal stability and multicolored emissions, these complexes were applied, for the first time, as selective ammunition markers, allowing for the unequivocal identification of the type of weapon used in forensic scenarios and the promotion of stricter ammunition trade control.

## 1 Introduction

The association of multidentate organic ligands with metal ions through electrostatic self-assembly results in the formation of porous coordination polymers. Among these porous materials, a particularly intriguing class is metal–organic frameworks, known as MOFs, which have garnered substantial interest within the scientific community.^1^ These materials received a provisional recommendation from the International Union of Pure and Applied Chemistry (IUPAC) in 2013, characterized as “a metal-organic network is a coordination polymer (or alternatively, a coordination network) presenting an open structure containing potential empty spaces”.^2^ These materials can be applied in various science fields, such as in gas adsorption^3^ and separation,^4^ drug delivery,^5^ catalysis,^6^ supercapacitors,^7^ and as electrochemical sensors.^8^

Some of these functionalities are possible due to inherent MOF porosity. Structural diversity can be generated through self-assembly between metal ions and organic ligands, where the inorganic unit (the metal ion) or the organic unit (the ligands) is constituted by mixtures of several metals or ligands. The use of a single metal ion for MOF production generates homometallic structures, which can be derived from both d-block^9^ and f-block metals, including lanthanides.^10,11^ The structural possibilities go beyond human imagination, allowing for the generation of compounds with extremely complex topologies, establishing an intimate structure–activity relationship.^12^ Furthermore, classical Lewis acid–base reactions also allow for the production of heterometallic MOFs of the 3d-4f type^13^ or even mixed MOFs containing different d-block^14^ or f-block metal fractions.^15^

Lanthanide metal–organic frameworks (LnMOFs) are obtained when lanthanide ions are used in the development of these materials, which are noteworthy for their diverse applications, mainly due to photophysical lanthanide ion properties.^16,17^ These polymeric networks can be obtained employing a variety of lanthanide ions, giving rise to mixed lanthanide metal–organic networks (m-LnMOFs).^18^ The possibility of tunable luminescence arouses significant interest in m-LnMOFs, whose emission intensities and colors can be adjusted by applying lanthanide ion mixtures during their synthesis.^12,19−21^ This light emission can be exploited to detect gunshot residues (GRS),^19^ a complex mixture of organic and inorganic materials produced during firearm firing.^22,23^ Some studies have proposed mixing luminescent markers to gun ammunition, as a simple portable UV radiation lamp would allow for the detection of luminescent gunshot residues (LGRS) at shooting sites.^24−31^ Specifically, LGSR can be easily detected on the ground, on the target, on spent cartridges, on weapons, and on the shooter’s hands, face, and clothing.^32^ Despite some studies carried out on luminescent firearm ammunition markers, the literature does not address such markers in long-range and high-energy firearms such as rifles. Additionally, no reports on the use of tunable luminescence m-LnMOFs as selective markers are available, which would allow for the visual distinction between different types of weapon ammunition or calibers. This application opens a range of possibilities, both in ammunition control and the standardization of different police force arsenals. In this sense, this study reports the synthesis, complete structural characterization, and photophysical property assessments of four new m-LnMOFs with the general formula [Eu_*x*_Tb_1–*x*_(Hbtec)]_*n*_ and [Eu_*x*_Tb_*y*_Gd_1–*x*–*y*_(Hbtec)]_*n*_. Due to their tunable luminescence, these compounds were tested as specific markers for four different types of firearms, namely, 0.380, 9, and 0.40 mm pistols and a 5.56 mm rifle.

## 2 Experimental Section

### 2.1 Materials and Measurements

The ligand used for the compound syntheses was 1,2,4,5-benzenetetracarboxylic acid (H_4_btec), and as salt, the terbium(III) chloride hexahydrate was used as purchased, without further purification. All reagents were purchased from Sigma-Aldrich Brasil. Europium and gadolinium metal salts (LnCl_3_.6H_2_O) were synthesized by the reaction of their respective oxides with concentrated hydrochloric acid (37% w/w), and the pH values were adjusted to about 6.0 by repeated evaporations. Structural characterizations were performed by Fourier transform near-infrared spectroscopy (FTIR), thermogravimetry (TG), powder X-ray diffraction (PXRD), scanning electron microscopy (SEM), and photoluminescence (PL) analyses. Inductively coupled plasma (ICP) spectroscopy assessments were performed to estimate the Eu^3+^/Tb^3+^ ratios of the mixed compounds, with samples prepared by digestion in concentrated HCl (37% m/m), followed by dilution to 0.5% HCl. All FTIR measurements were performed in the ATR mode, with spectra obtained in the 4000–500 cm^–1^ range employing a PerkinElmer Spectrum One instrument, applying an average of 128 scans at a spectral resolution of 4 cm^–1^. The TG curves were obtained from 50 to 800 °C, using 3 mg of each compound and applying a heating rate of 10 °C/min under a nitrogen atmosphere employing a Q50 apparatus (TA Instruments, USA). PXRD measurements were performed using a D8 ADVANCE diffractometer, set at a 45 kV tube voltage, 40 mA current, and CuKα radiation in Bragg–Brentano geometry. The analyses were conducted at 2θ ranging from 7 to 70 °C and at a step angle of 0.01° for 1 h. The photophysical study was carried out by using a Jobin-Yvon Fluorolog-322 spectrofluorimeter equipped with an R928 Hamamatsu photomultiplier and a 450 W xenon lamp excitation source. This equipment contains a TRIAX 320 double monochromator for both excitation and emission measurement. The morphologies of the synthesized compounds were investigated by SEM employing a JSM7100FT JEOL SEM instrument at an accelerating voltage of 15 kV.

### 2.2 Synthesis of the [Eu_*x*_Tb_1–*x*_Hbtec]_*n*_ and [Eu_*x*_Tb_*y*_Gd_1–*x*–*y*_Hbtec]_*n*_ Series

**Scheme 1:**
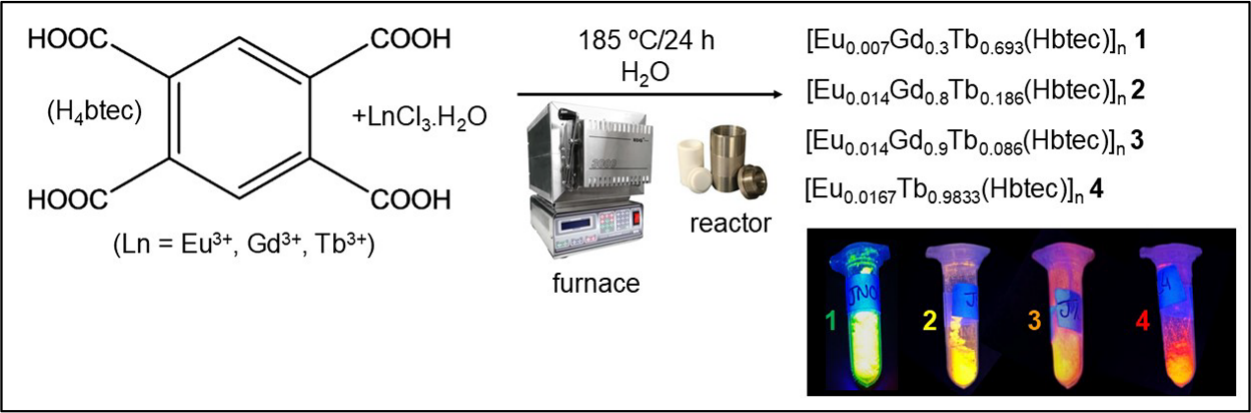
Synthetic Route for m-LnMOFs and Their Tunable Luminescence When Irradiated at λ = 254 nm

The syntheses of both m-LnMOFs series were performed by the hydrothermal method (Scheme 1), following a procedure reported for previous m-LnMOFs^33^ and homometallic [Tb(Hbtec)]_*n*_^29^ and [Eu(Hbtec)]_*n*_^31^ studies. The mixed frameworks were synthesized by varying the original EuCl_3_.6H_2_O, GdCl_3_.6H_2_O, and TbCl_3_.6H_2_O molar ratios through the same synthetic procedures. The synthesis of the mixed compound [Eu_0.007_Gd_0.3_Tb_0.693_(Hbtec)]_n_**1** is detailed below.

#### 2.2.1 Synthesis of [Eu_0.007_Gd_0.3_Tb_0.693_(Hbtec)]_*n*_**1**

A mixture of H_4_btec acid (0.100 g, 0.393 mmol), EuCl_3_.6H_2_O (1.008 mg, 0.003 mmol), TbCl_3_·6H_2_O (101.7 mg, 0.272 mmol), and GdCl_3_.6H_2_O (43.8 mg, 0.118 mmol) was dissolved in 20 mL of distilled water and stirred for 3 h at room temperature. The resulting mixture was then heated at 185 °C for 3 days in a 50 mL Teflon-lined stainless steel vessel under autogenous pressure. Subsequently, the reaction mixture was slowly cooled to room temperature for 24 h. Yield: 59.36%. Anal. calc. for C_10_H_3_O_8_Eu_0.007_Gd_0.3_Tb_0.693_: %C: 29.30, %H: 0.73; found: %C: 30.01, %H: 0.74. *m*_obtained_: 0.0955 g.

#### 2.2.2 Synthesis of [Eu_0.014_Gd_0.8_Tb_0.186_(Hbtec)]_*n*_**2**

The same synthetic procedure adopted for compound **1** was used for compound **2**, except for the amounts of EuCl_3_.6H_2_O (2.016 mg, 0.0055 mmol), GdCl_3_.6H_2_O (116.8 mg, 0.314 mmol), and TbCl_3_.6H_2_O(27.2 mg, 0.073 mmol). Yield: 51.95%. Anal. calc. for C_10_H_3_O_8_Eu_0.014_Gd_0.8_Tb_0.186_: %C: 29.36, %H: 0.73; found: %C: 29.40, %H: 0.73. *m*_obtained_: 0.0834 g.

#### 2.2.3 Synthesis of [Eu_0.014_Gd_0.9_Tb_0.086_(Hbtec)]_*n*_**3**

The same synthetic procedure adopted for compound **1** was used for compound **3**, except for the amounts of EuCl_3_.6H_2_O (2.016 mg, 0.0055 mmol), GdCl_3_.6H_2_O (131.4 mg, 0.354 mmol), and TbCl_3_.6H_2_O (12.62 mg, 0.033 mmol). Yield: 54.59%. Anal. calc. for C_10_H_3_O_8_Eu_0.014_Gd_0.9_Tb_0.086_: %C: 29.37, %H: 0.73; found: %C: 29.99, %H: 0.72. *m*_obtained_: 0.0876 g.

#### 2.2.4 Synthesis of [Eu_0.0167_Tb_0.9833_(Hbtec)]_*n*_**4**

The same synthetic procedure adopted for compound **1** was used for compound **4**, except for the amounts of EuCl_3_.6H_2_O (2.405 mg, 0.006 mmol) and TbCl_3_.6H_2_O (144.2 mg, 0.386 mmol). Yield: 58.36%. Anal. calc. for C_10_H_3_O_8_Eu_0.0167_Tb_0.9833_: %C: 29.27, %H: 0.73; found: %C: 30.02, %H: 0.74. *m*_obtained_: 0.0940 g.

All LnMOFs were properly washed with 2 portions (10 mL) of hot water to remove possible impurities. Such compounds were obtained as white solids, with good yields and high crystallinity degrees. The Eu^3+^/Gd^3+^/Tb^3+^molar ratios in compounds **1**, **2,** and **3** and that of Eu^3+^/Tb^3+^ in compound **4** matched the original lanthanide cation molar ratios in the starting chemicals. These results were confirmed by ICP-MS analyses (Table S1, in Supporting Information). Additionally, all synthesized m-LnMOFs presented tunable luminescence, with[Eu_0.007_Gd_0.3_Tb_0.693_(Hbtec)]_*n*_**1** emitting in the green region, [Eu_0.014_Gd_0.8_Tb_0.186_(Hbtec)]_*n*_**2** emitting in the yellow region, [Eu_0.014_Gd_0.9_Tb_0.086_(Hbtec)]_*n*_**3** emitting in the orange region, and[Eu_0.0167_Tb_0.9833_(Hbtec)]_*n*_**4** emitting in the red region (Scheme 1).

It is important to note the essential role of Gd^3+^ in compounds **1**–**3**, which, despite not emitting radiation in the visible spectrum, plays a role in diluting Eu^3+^ and Tb^3+^ ion concentrations in the polymer networks, achieving tunable luminescence. In contrast, compound **4** contains only Eu^3+^ and Tb^3+^ ions, intensely emitting in the red region, due to the Tb^3+^ → Eu^3+^ energy transfer.

### 2.3 Selective Gunshot Residue(GSR) Marker Tests

**Figure 1:**
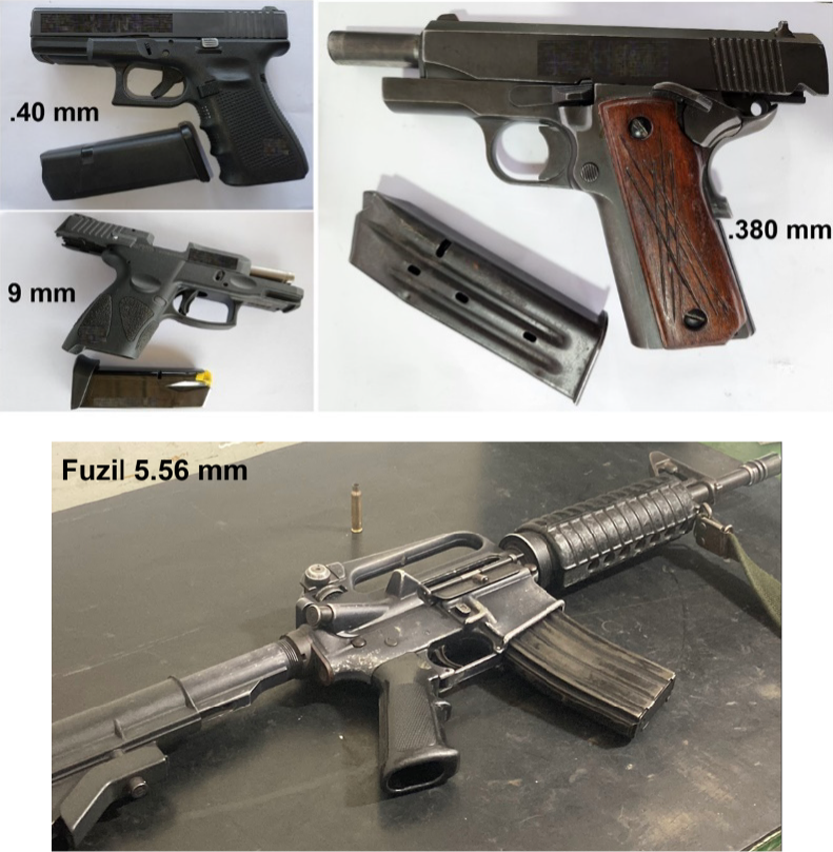
Weapons used in the applied ballistics tests: Imbel GC MD1 pistol, 0.380 mm caliber; Taurus G2c pistol, 9 mm caliber; Glock G23 pistol, 0.40 mm caliber; and Taurus T4 rifle, 5.56 mm caliber.

Tests involving the synthesized m-LnMOFs as ammunition markers were carried out at a Civil Police of the state of Rio de Janeiro (PCERJ) training area. The location and employed weapons were provided by the Civil Police. An inertia hammer was used to open the firearm ammunition and insert the synthesized compounds. The marker, a white powder under normal light, was then mixed with the gunpowder. The projectiles were then rejoined with their cartridges. A target located 2 m from the shooter was used, with the target and the ground covered with black TNT fabric to check for the residues. The target was made of cardboard and was changed after three shots with each specific firearm. A device called a chronograph placed between the shooter and the target was used to measure ballistic feet per second (FPS), which can be converted to m s^–1^ and allows for the calculation of projectile speed. The FPS of a 5.56 mm rifle projectile was measured to determine the bullet's influence on projectile ejection speed. The shooters involved in the tests were suggested by the PCERJ team. Four different weapons were used, namely, an Imbel GC MD1 pistol, 0.380 mm caliber; a Taurus G2c pistol, 9 mm caliber; a Glock G23, 0.40 mm caliber; and a Taurus T4 rifle, 5.56 mm caliber (see Figure 1). About 40–50 mg of tracer was added to all pistols, distributed as follows: ([Eu_0.014_Gd_0.9_Tb_0.086_(Hbtec)]_*n*_**3** in the 0.380 mm pistol; [Eu_0.0167_Tb_0.9833_(Hbtec)]_*n*_**4** in the 9 mm pistol; and [Eu_0.014_Gd_0.8_Tb_0.186_(Hbtec)]_*n*_**2** in the 0.40 mm pistol. Concerning the rifle, for safety reasons, as the amount of propellant charge is high, about 0.1949 g of gunpowder was removed, and three different amounts (180, 250, and 350 mg) of [Eu_0.007_Gd_0.3_Tb_0.693_(Hbtec)]_*n*_**1** were added.

## 3 Results and Discussion

### 3.1 Infrared Vibrational Spectroscopy Analyses

Table S1 illustrates the main vibrational bands in the infrared region of the previously reported sodium salt Na_4_btec, the acid H_4_btec, the homometallic networks [Tb(Hbtec)]_*n*_^29^ and[Eu(Hbtec)]_*n*_^31^ and the four synthesized m-LnMOFs. Figure S1 (Supporting Information) displays the respective absorption spectra for 1,2,4,5-benzenetetraboxylic acid and its respective sodium salt, and Figure S2 (Supporting Information) displays the vibrational spectra for compounds **1**–**4**. Stretching bands ν(C–H) and ν(C=C) attributed to the aromatic portion of the Hbtec^3–^ligand are observed. Furthermore, ν(C=O) and ν(O–H) stretching bands characteristic of carboxylic groups were also observed, indicating that at least one of these groups is not deprotonated. One of the most important data obtained from vibrational spectroscopy is associated with the coordination modes of carboxylate groups. According to Deacon,^34^ such coordination modes can be inferred by comparing the parameter Δ = [ν_asym_(COO^–^) – ν_sym_(COO^–^)] of the respective precursor sodium salt and the synthesized compounds. The obtained Na_4_btec value was 189 cm^–1^. Two symmetric stretching modes were assigned to the four m-LnMOFs (ν_sym_COO^–^ ∼ 1373 and 1463 cm^–1^) as well as an asymmetric stretching mode (ν_asym_COO^–^ ∼ 1613 cm^–1^), resulting in two Δ values (240 and 150 cm^–1^), suggesting monodentate and bidentate chelate coordination modes of the COO^–^ groups to the metal centers (see Table S2, in Supporting Information).

### 3.2 Thermal Analysis (TG Curves)

**Figure 2:**
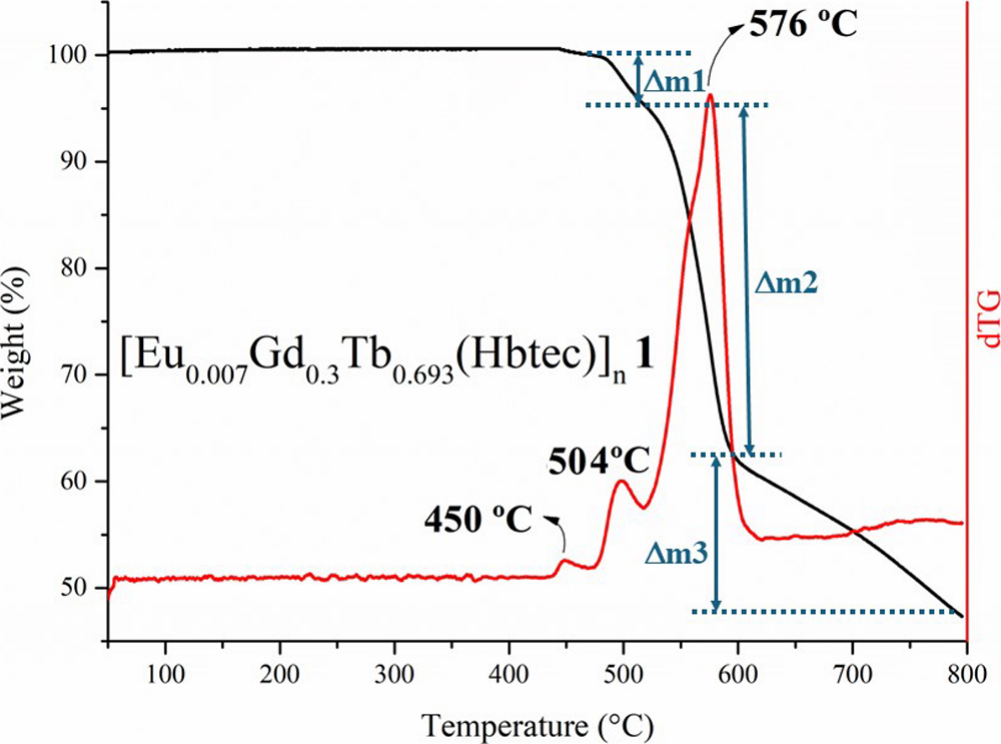
TG (black) and dTG (red) curves for [Eu_0.007_Gd_0.3_Tb_0.693_(Hbtec)]_*n*_**1** obtained under a N_2_ atmosphere.

The main objective of the thermal analysis in this work is to verify the high thermal stability of the synthesized compounds since, during the firing of a firearm, the temperature can reach high temperatures. Analyzing the TG curve (in black) for compound [Eu_0.007_Gd_0.3_Tb_0.693_(Hbtec)]_*n*_**1** (Figure 2), no weight loss related to water molecules is observed (in the range 25–450 °C), indicating the absence of water in the structure, with this observation corroborated by results of vibrational spectroscopy.

The compound starts to decompose at 450 °C, and above this temperature occurs the first loss mass being attributed to the release of a carbon monoxide molecule (obsd 6.71%; calc. 6.68%). Two consecutive mass losses (obsd 41.05%) correspond to the loss of the ligand via the generation of a benzene derivative (calcd 42.02%), with a peak at 576 °C (indicated in the dTG curve). Residue formation takes place at temperatures higher than 800 °C, as observed for the isostructural homometallic compound [Eu(Hbtec)]_*n*_,^29^ which is formed by carbonized material from the organic ligand and a complex mixture of oxides of the respective lanthanides. The formation of these oxides was observed through X-ray diffraction data obtained at 800 °C, as discussed below. The literature reports a very similar thermal behavior for compounds with the general formula [Ln(Hbtec)(H_2_O)] (Ln = Pr, Eu, and Gd).^35^ As expected in a series of isostructural compounds, the other synthesized m-LnOFs presented the same thermal behavior observed for **1**. Their respective TG curves are reported in Figures S2–S4 (Supporting Information).

### 3.3 PXRD Analyses

**Figure 3:**
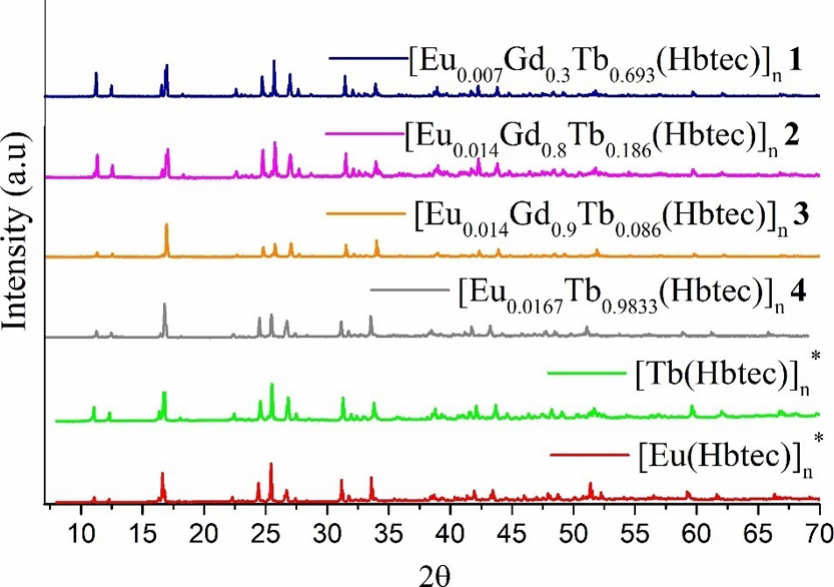
X-ray diffractograms patterns for all synthesized m-LnOFs (**1**–**4**). Asterisks indicate the diffractograms of previously synthesized isostructural homometallic MOFs.

X-ray diffraction measurements through polycrystals revealed that all m-LnOFs are isostructural, also isostructural to the previously synthesized homometallic MOFs [Tb(Hbtec)]_*n*_,^29^ [Eu(Hbtec)]_*n*_^31^ and with determined crystal structures (see Figure 3). Thus, all m-LnMOFs reported herein crystallize in the triclinic system and with space group *P*1. A Pawley refinement was performed for all m-LnMOFs, allowing for the determination of the unit cell and refinement quality parameters for compounds **1**–**4**. Furthermore, the volume of each unit cell of the synthesized m-LnMOFs was also investigated, allowing for the determination of the influence of the insertion of different lanthanide ion fractions in their primitive unit cell volumes, as depicted in Table S3, in Supporting Information. The ionic radii for each lanthanide ion are *r*(Eu^3+^) = 1.12 Å, *r*(Gd^3+^) = 1.107 Å, *r*(Tb^3+^) = 1.095 Å, respectively.^36,37^ These data indicate that the primitive unit cell volume for compound [Eu_0.007_Gd_0.3_Tb_0.693_(Hbtec)]_*n*_**1** is 456.51 Å^3^. When comparing compound [Eu_0.014_Gd_0.8_Tb_0.186_(Hbtec)]_*n*_**2** to [Eu_0.014_Gd_0.9_Tb_0.086_(Hbtec)]_*n*_**3**, the cell unit volume of the latter is larger, due to the more abundant Gd^3+^ ion fraction. In this case, the Eu^3+^fraction is the same, with variations noted only for the Gd^3+^and Tb^3+^ion concentrations and, obviously, the increase in unit cell volume due to the more abundant Gd^3+^ ion fraction, with a greater ionic radius in relation to Tb^3+^. It is clear that the unit cell volume is, thus, susceptible to changes in lanthanide ion fractions due to different lanthanide ionic radii.

**Figure 4:**
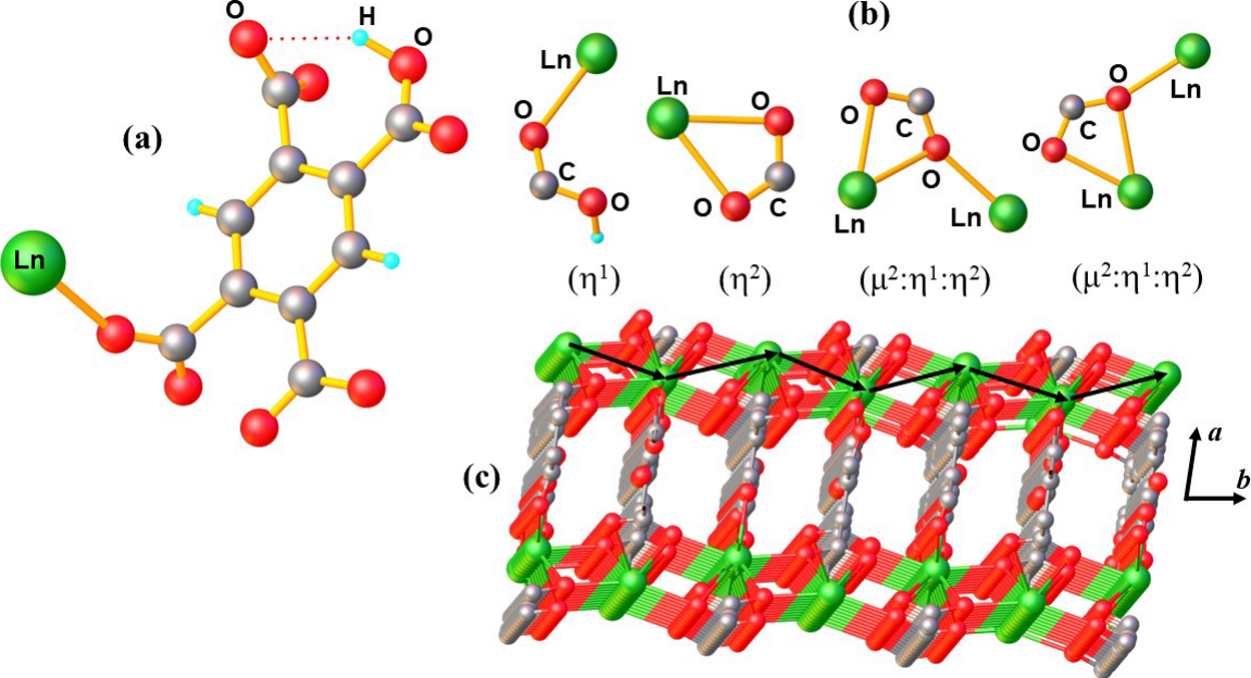
(a) Asymmetric unit for the formed mixed lanthanide metal–organic frameworks. (b) Coordination modes of the carboxylate groups in compounds. (c) 3D network for the synthesized mixed lanthanide metal–organic frameworks, evidencing the zigzag chain formed by SBUs (black arrows).

X-ray diffraction measurements were also performed applying temperature variations from 25 to 1000 °C. Figure S4 (Supporting Information) displays all obtained diffractograms. Similar degradation profiles are observed with varying medium temperatures, corroborating the TG curves for all structures (**1**–**4**). These data also reveal that the diffraction profile between 100 and 400 °C is similar to that observed at 25 °C, demonstrating that the investigated polymer networks do not collapse up to 400 °C. A clear change in the diffraction profiles is noted at 500 °C, indicating the collapse of the mixed lanthanide networks, once again in agreement with the TG curves. Finally, the diffraction profiles observed between 800 and 1000 °C are similar to those of europium oxide (Eu_2_O_3_), gadolinium oxide (Gd_2_O_3_), and one of the main terbium oxides (Tb_2_O_3_). Therefore, only a mixture of lanthanide oxides is present in the system above 800 °C. As discussed previously, the m-LnOFs synthesized herein are isostructural to their homometallic counterparts. [Tb(Hbtec)]_*n*_^29^ and [Eu(Hbtec)]_*n*_,^31^ whose crystal structures were determined. Figure 4a displays the asymmetric unit of these compounds formed by a Hbtec^3–^anion and a Ln^3+^ion.

The Hbtec^3–^ligand contains four carboxylate groups, acting in three different coordination modes to the metal centers, namely, two COO^–^groups inμ_2_:η^1^:η^2^ (chelate/bridge), one COO^–^group in η^1^ (monodentate), and one in η^2^ (chelate), as observed in Figure 4b. A zigzag-shaped chain (black arrows) is formed by [LnO_9_] units, classified as well-known MOF SBUs.^31^ These SBUs connect simultaneously through carboxylate groups, giving rise to a 3D polymeric structure (Figure 4c).

### 3.4 SEM Analyses

**Figure 5:**
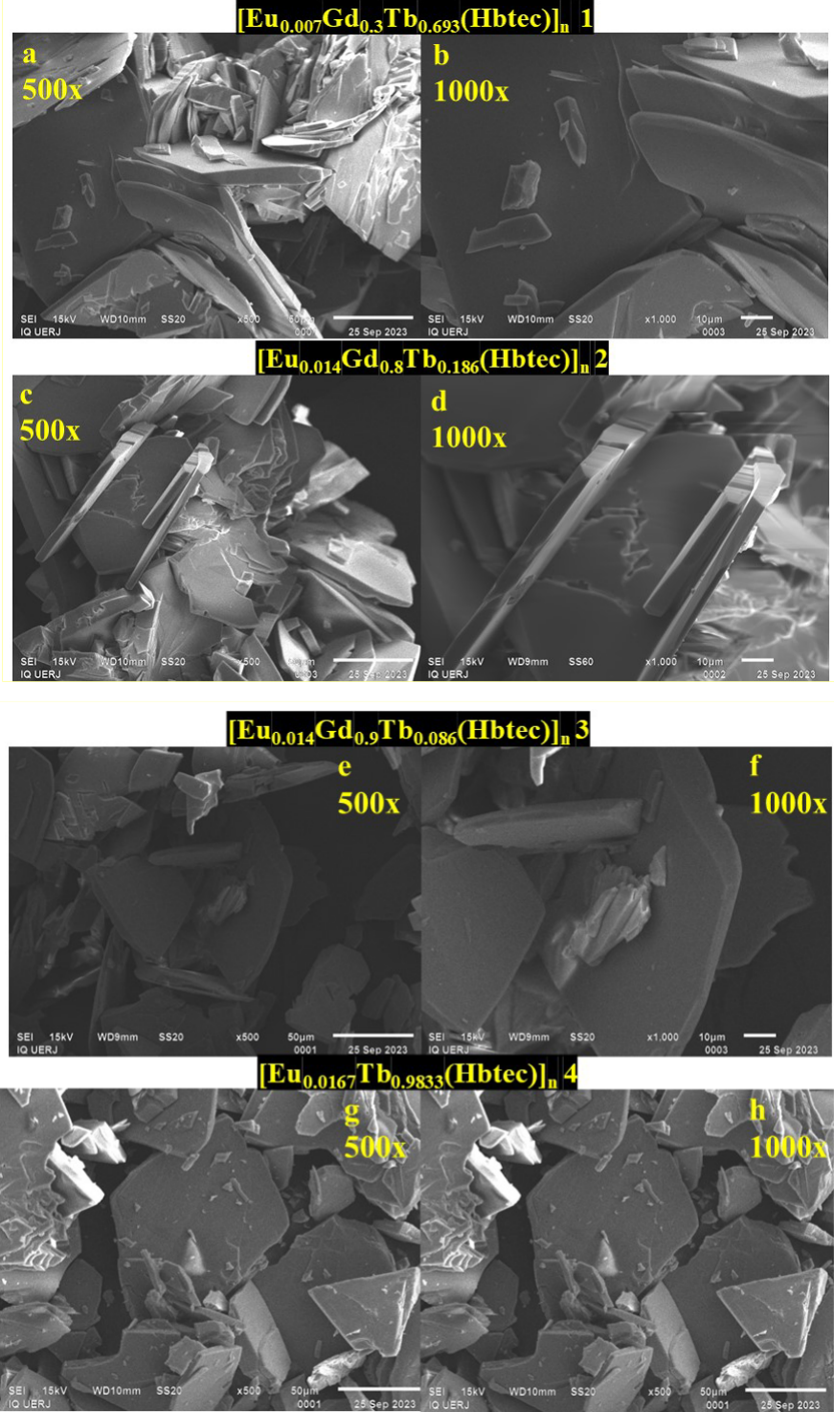
Scanning electron microscopy (SEM) images for [Eu_0.007_Gd_0.3_Tb_0.693_(Hbtec)]_*n*_**1** (a, b); [Eu_0.014_Gd_0.8_Tb_0.186_(Hbtec)]_*n*_**2** (c, d); [Eu_0.014_Gd_0.9_Tb_0.086_(Hbtec)]_*n*_**3** (e, f); [Eu_0.0167_Tb_0.9833_(Hbtec)]_*n*_**4** (g, h) of the synthesized mixed lanthanide metal–organic frameworks. On the left, 500× magnification; to the right, 1000× magnification.

Images were obtained using an SEM apparatus to evaluate microcrystal morphology changes depending on the fraction of each lanthanide ion. The results are depicted in Figure 5a,b for compound [Eu_0.007_Gd_0.3_Tb_0.693_(Hbtec)]_*n*_**1**; 5c and 5d for compound [Eu_0.014_Gd_0.8_Tb_0.186_(Hbtec)]_*n*_**2**; 5e and 5f for compound [Eu_0.014_Gd_0.9_Tb_0.086_(Hbtec)]_*n*_**3**, 5g and 5h for compound [Eu_0.0167_Tb_0.9833_(Hbtec)]_*n*_**4**, at 500× (left) and 1000× (right) magnification. The analyzed microcrystals present a structural morphology in the form of juxtaposed plates for all analyzed samples and also indicate that altering lanthanide ion concentrations does not directly interfere with m-LnMOF microcrystal morphology.

### 3.5 PL Study

**Figure 6:**
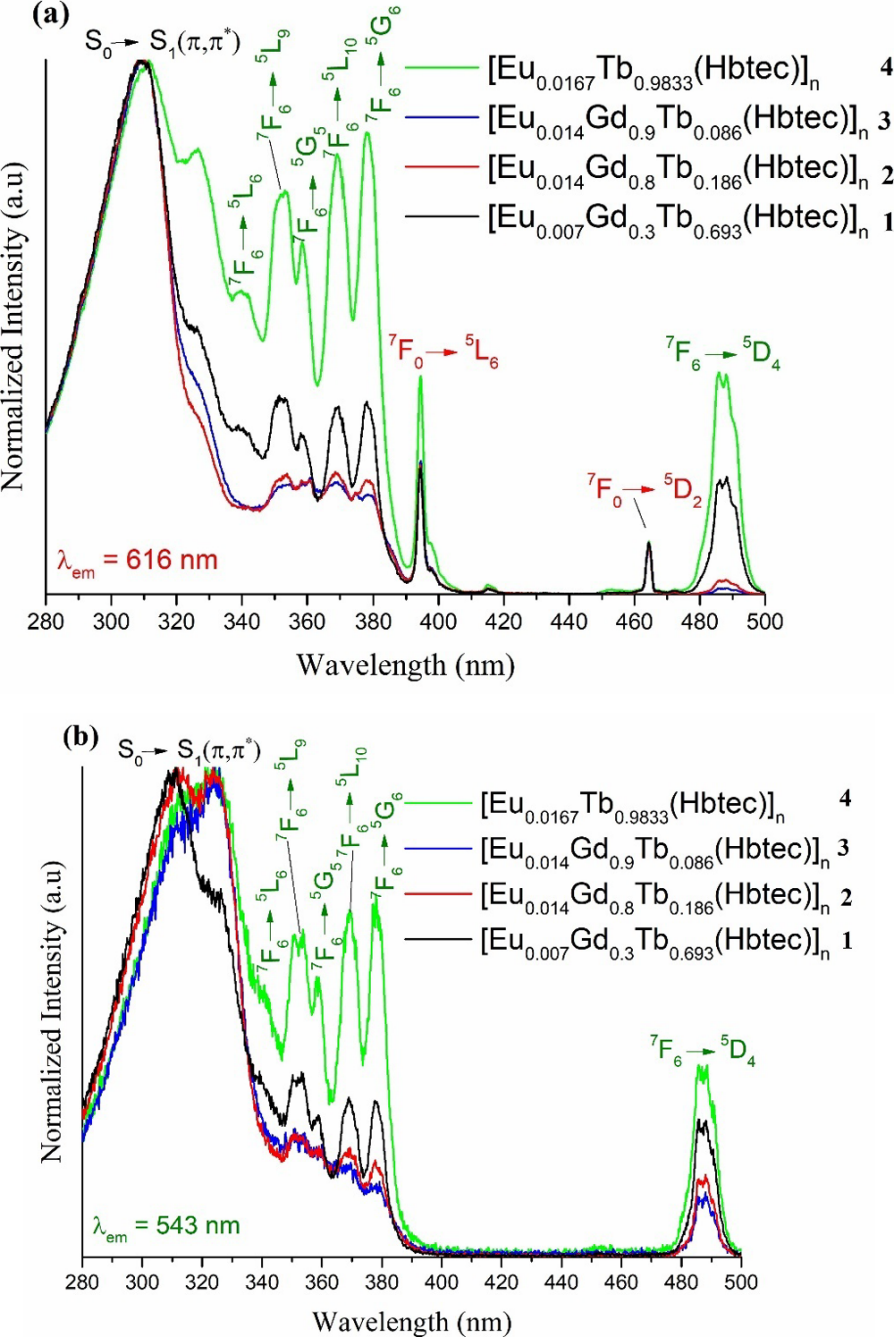
(a) Excitation spectra of all synthesized compounds at room temperature (**1**–**4**) with emissions monitored at 616 and (b) 543 nm.

Excitation spectra obtained at room temperature and in the 280–500 nm range are displayed in Figure 6a,b, illustrating absorption band profiles, with emissions monitored at 616 nm (centered on the Eu^3+^ ion, Figure 6a) and 543 nm (centered on the Tb^3+^ ion, Figure 6b) for the four investigated compounds. Bands attributed to the S_0_ → S_1_ transition related to the Hbtec^3–^ ligand are observed in the 280–335 nm range in all excitation spectra. This broadband, which is more intense than 4f-4f transitions, reveals that the employed ligand is efficient in sensitizing the lanthanide ions present in the synthesized m-LnMOFs.The characteristic intraconfigurational transitions of Eu^3+^ are highlighted in red, specifically ^7^F_0_ → ^5^L_6_ e ^7^F_0_ → ^5^D_2_ at 395 and 463 nm, respectively. In addition, the transitions related to Tb^3+^ are marked in green, as follows: ^7^F_6_ → ^5^L_6_ (340 nm), ^7^F_6_ → ^5^L_9_ (353 nm), ^7^F_6_ → ^5^G_5_ (358 nm), ^7^F_6_ → ^5^L_10_ (369 nm), ^7^F_6_ → ^5^G_6_ (378 nm), and ^7^F_6_ → ^5^D_4_ (487 nm). It is interesting to note that the electronic transitions attributed to the Eu^3+^ ion are only present in the excitation spectrum with an emission wavelength fixed at 616 nm. Such transitions centered on the Eu^3+^ ion only in the spectra with λ_em_ = 616 nm may indicate a Tb^3+^ → Eu^3+^ energy transfer. A direct relationship between lanthanide ion fraction modifications and intensity variations of the 4f-4f bands was not possible to trace in these spectra.

**Figure 7:**
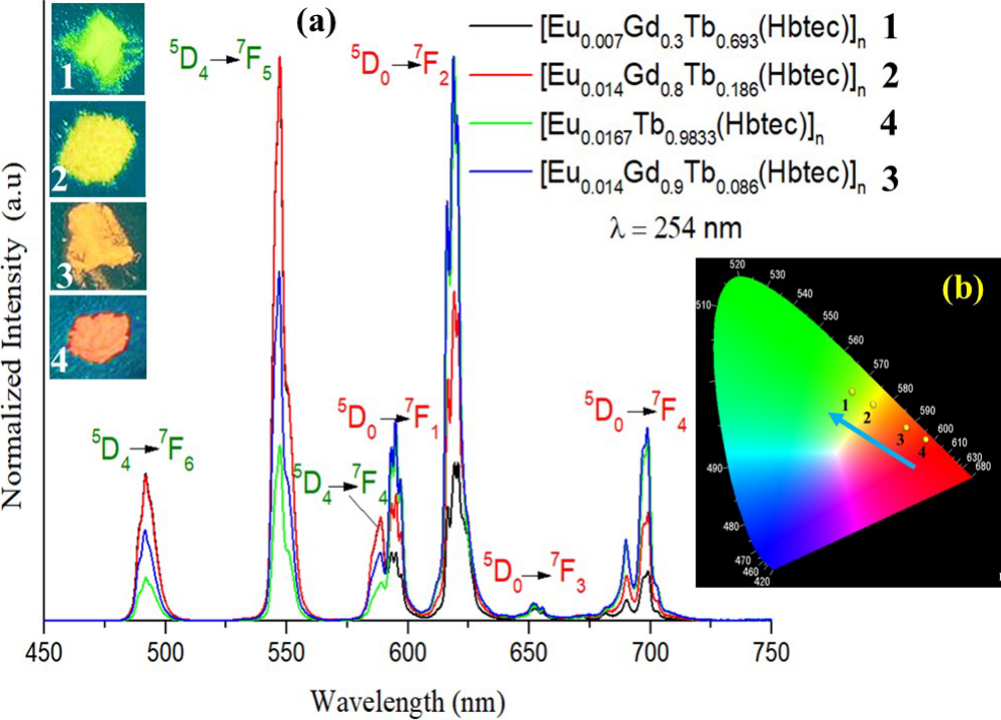
(a) Emission spectra for **1**–**4** mixed lanthanide metal–organic frameworks under excitation at 254 nm, illustrating their tunable luminescence. (b) CIE diagram (obtained from Spectra Lux software)^39^presenting chromaticity coordinate shifting.

Figure 7a displays the emission spectra obtained between 450 and 750 nm under excitation for the greatest ligand absorption intensity band observed at 254 nm.

The 4f-4f transitions for all m-LnOFs are centered in the same spectrum region, although with intensities varying according to the molar fraction of each of the lanthanide ions present in the synthesized structures. The spectra reveal thin emission bands from Eu^3+^ (in red): ^5^D_0_ →^7^F_1_ (593 nm), ^5^D_0_ → ^7^F_2_ (619 nm), ^5^D_0_ → ^7^F_3_ (653 nm), and^5^D_0_ → ^7^F_4_ (696 nm) and from Tb^3+^ (in green): ^5^D_4_ → ^7^F_6_ (491 nm), ^5^D_4_ → ^7^F_5_ (546 nm), e ^5^D_4_ → ^7^F_4_ (588 nm), and ^5^D_4_ → ^7^F_4_ (588 nm).

The appearance of transitions for both ions (Eu^3+^ and Tb^3+^) once again suggests the incorporation of both ions into the mixed lattices. The emission spectra demonstrate that the transition intensities centered on the Tb^3+^ ion for compounds [Eu_0.007_Gd_0.3_Tb _0.693_(Hbtec)]_*n*_**1** and [Eu_0.014_Gd_0.8_Tb_0.186_(Hbtec)]_*n*_**2** are more intense than the characteristic Eu^3+^ transitions. This intensity profile is also corroborated by the CIE diagram (Figure 7b, obtained from Spectra Lux software),^38^ observing tunable luminescence, i.e., a green emission for compound [Eu_0.007_Gd_0.3_Tb_0.693_(Hbtec)]_*n*_**1** (CIE coordinates: *x* = 0.394 and *y* = 0.509), while compound [Eu_0.014_Gd_0.8_Tb_0.186_(Hbtec)]_*n*_**2** emits in the yellow region, with CIE coordinates *x* = 0.454 and *y* = 0.470. In contrast, the emission profiles for [Eu_0.014_Gd_0.9_Tb_0.086_(Hbtec)]_*n*_**3** and [Eu_0.0167_Tb_0.9833_(Hbtec)]_*n*_**4** are the opposite, with the electronic transitions originating from the Eu^3+^ ion being the most intense. Therefore, the colors emitted by these last two compounds are located in the orange region for [Eu_0.014_Gd_0.9_Tb _0.086_(Hbtec)]_*n*_**3** (CIE coordinates: *x* = 0.548 e *y* = 0.406) and red for [Eu_0.0167_Tb _0.9833_(Hbtec)]_*n*_**4** (CIE coordinates: *x* = 0.601 and *y* = 0.370). The color shift from green to red is possibly due to Tb^3+^ → Eu^3+^ energy transfer. It is also important to note that our group has reported a series of m-LnMOFs with the general formula [Eu_*x*_Tb_1–*x*_Hbtec]_*n*_ (0.1 ≤ *x* ≤ 0.9),^33^ although only compounds that emit in different red regions were obtained with the applied molar fractions and types of Ln^3+^ ions. Concerning the mixed MOFs containing Gd^3+^, these cations increase the distance between the Eu^3+^ and Tb^3+^ ions, decreasing the probability of a Tb^3+^ → Eu^3+^ energy transfer, enabling emissions closer to the green CIE region, such as the yellow emission verified by compound [Eu_0.014_Gd_0.8_Tb_0.186_(Hbtec)]_*n*_**2**. Other strategies can be used to obtain yellow emissions in MOFs, such as doping with Eu^3+^ and Tb^3+^ ions in a d–f heterometallic MOF, with the formula [H(H_2_O)_8_LaZn_4_(4,5-imidazole-dicarboxylate)_4_(imidazole)_4_].^39^ In another example, Wang and co-workers^40^ also used a tetracarboxylic acid (5,5′-oxidiisophthalic acid) to construct m-LnMOFs 1-Eu_*x*_Tb_*y*_, which display multicolored emissions at different green and yellow scales. Furthermore, the absence of the transition relative to the Hbtec^3–^ ligand in the emission spectra suggests an efficient ligand energy transfer → (Eu^3+^, Tb^3+^). The triplet energy level (T_1_) of the Hbtec^3–^ ligand is relatively high ∼25.675 cm^–133^, being able to sensitize both Eu^3+^ (^5^D_1_ ∼ 19.000 cm^–1^ and ^5^D_0_ ∼ 17.300 cm^–1^) and Tb^3+^ (^5^D_3_ ∼ 25.700 cm^–1^ and ^5^D_4_ ∼ 20.400 cm^–1^).

#### 3.5.1 Decay Lifetimes

All decay curves of the emitting states were fitted using a monoexponential decay model (Figure S3, Supporting Information). The lifetime curves of the emitting Eu^3+^ and Tb^3+^ ion states presented distinct values in each compound, as expected. The homometallic isostructural MOFs [Tb(Hbtec)]_*n*_,^29^ [Eu(Hbtec)]_*n*_^31^ display lifetime values of τ(^5^D_4_) = 0.75 ± 0.06 ms and τ(^5^D_0_) = 1.22 ± 0.01 ms, respectively. Two lifetime values were obtained for the synthesized m-LnMOFs, corresponding to the emitting states of Eu^3+^ (^5^D_0_) and Tb^3+^ (^5^D_4_). Table S4 (in Supporting Information) depicts the decay values of the emitting states for all the investigated m-LnMOFs, while Figure S5a–h (Supporting Information) show the fit curves of their respective lifetimes.

For m-LnMOFs containing Eu^3+^ and Tb^3+^, the energy transfer efficiency Tb^3+^ → Eu^3+^ can be calculated using the equation η_ET_ = (1 – τ/τ_0_), where τ and τ_0_ represent the decay times of the Tb^3+^ ion with and without the energy acceptor (Eu^3+^ ion), respectively.^33^ Thus, considering the mixed m-LnMOFs previously studied by our group, [Eu_0.1_Tb_0.9_(Hbtec)]_*n*_^33^ and compound [Eu_0.0167_Tb_0.9833_(Hbtec)]_*n*_**4**, the determined η_ET_ values are 98% for the first compound and 38% for **4**. In this case, the transfer efficiency Tb^3+^ → Eu^3+^ is considerably greater for that compound, with the highest Eu^3+^/Tb^3+^ quotient value, as expected. This is corroborated by the intense red region emission observed for [Eu_0.1_Tb_0.9_(Hbtec)]_*n*_ (CIE coordinates *x* = 0.666 and *y* = 0.327) compared to a lighter shade of red for [Eu_0.0167_Tb_0.9833_(Hbtec)]_*n*_**4** (CIE coordinates *x* = 0.601 and *y* = 0.370). An interesting fact is that the lifetimes of m-LnMOFs containing Gd^3+^ are longer than those observed for their homometallic counterparts. This may be due to an increase in the distance between the Eu^3+^ and Tb^3+^ ions, caused by the insertion of Gd^3+^ in the synthesized networks, consequently increasing their decay time values. Obviously, the number of studied homometallic LnMOFs is much higher than that of their mixed analogues. However, the literature reports different interesting studies on m-LnMOFs achieving tunable luminescence. In one of them, Du and co-workers report a series of examples of mixed lanthanide networks employing polytopic carboxylate ligands.^41^

### 3.6 m-LnMOFs as Selective Ammunitions Markers

Ammunition marking tests were conducted using four different types of weapons, namely, one 0.380 mm GC MD1 Imbel pistol; one 9 mm G2c Taurus pistol; one 0.40 mm G23 Glock pistol, and one 5.56 mm T4 Taurus rifle (see Figure 1). All weapons were spiked with about 40–50 mg of tracers, ([Eu_0.014_Gd_0.9_Tb_0.086_(Hbtec)]_*n*_**3** in the 0.380 mm pistol; [Eu_0.0167_Tb_0.9833_(Hbtec)]_*n*_**4** in the 9 mm pistol; and [Eu_0.014_Gd_0.8_Tb_0.186_(Hbtec)]_*n*_**2** in the 0.40 mm pistol. In the rifle, for safety reasons, since the amount of propellant charge is high, about 0.1949 g of gunpowder was removed and three different amounts (180, 250, and 350 mg) of [Eu_0.007_Gd_0.3_Tb_0.693_(Hbtec)]_*n*_**1** were added. Initially, it is important to note that the purpose of our study was to apply tunable luminescence to distinguish between different types of ammunition (or weapon calibers). To the best of our knowledge, no reports on the use of tunable luminescence in m-LnMOFs as a proposal to differentiate different types of ammo are available. Additionally, the literature does not report marking tests on ammunition used in long-range and high-energy weapons such as rifles. Therefore, this study is paramount in the Public Security area, allowing for stricter ammunition marketing control, for example.

**Figure 8:**
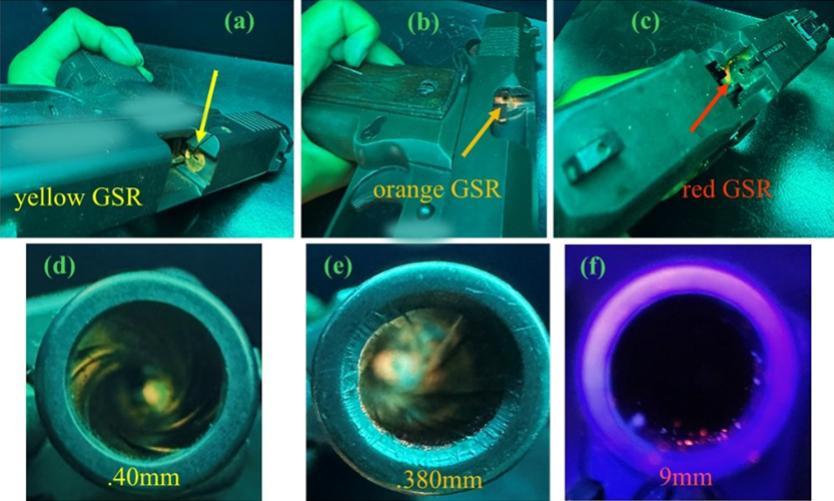
(a), (b), and (c)–yellow, orange, and red luminescent markers in ejection port/extractors. (d), (e), and (f)–yellow, orange, and red GSR detected inside the weapon muzzles, with the helical grooves observed in the 0.40 mm Glock G23, 0.380 mm ImbelGC MD1and 9 mm Taurus G2c pistols. The luminescent GSR were detected by irradiation at λ = 254 nm.

After three consecutive shots with the aforementioned weapons, selected m-LnMOFs formed LGSRin in different firearm compartments (Figure 8). Furthermore, the LGSR was very evident in the ejection ports/extractor of the three pistols, specifically a yellow gunshot residue in the 0.40 mm Glock G23 pistol (Figure 8a), an orange gunshot residue in the 0.380 mm Imbel pistol (Figure 8b), and a red gunshot residue in the 9 mm Taurus G2c pistol (Figure 8c). These same markers were very efficient in marking the helical grooves contained inside the barrels of the 0.40 mm (Figure 8d), 0.380 mm (Figure 8e), and 9 mm Taurus G2c pistol (Figure 8f) pistols.

**Figure 9:**
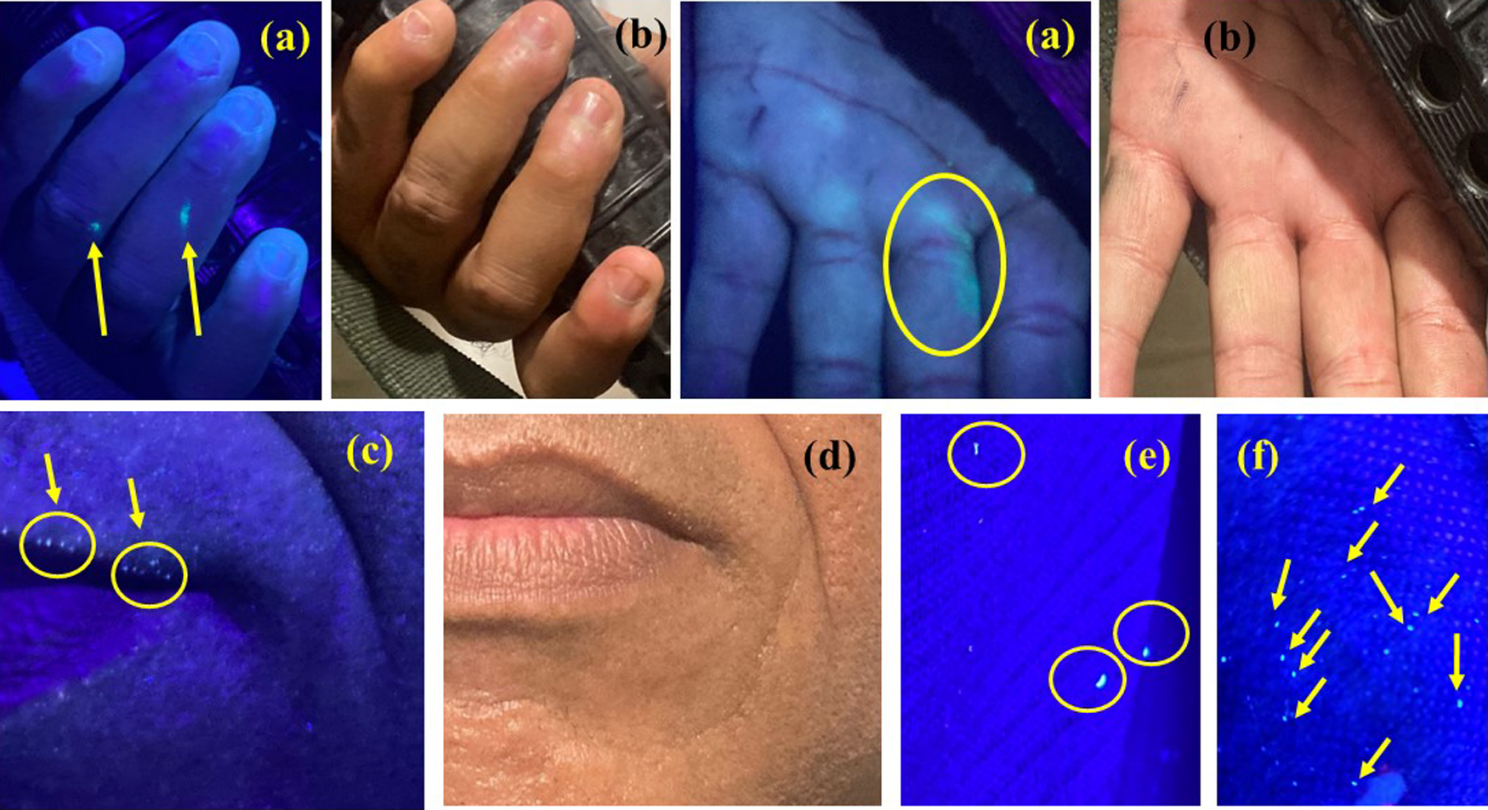
(a) Shooter’s hands under UV light (λ = 254 nm); (b) shooter’s hands under normal light; (c) shooter’s lips with green GSR under UV light; (d) shooter’s lips with under normal light; (e) shooter shirt with green GSR under UV light; and (f) floor under shooting with several green GSR at λ = 254 nm.

Green luminescent particles were observed under UV light illumination (λ = 254 nm) after three shots with the 5.56 mm Taurus T4 rifle on the shooter’s hands, lips, and shirt. Figure 9a shows the shooter’s hand irradiated with UV light, while Figure 9b shows the same photographs under normal light. Figure 9c,e shows the shooter’s lips and a portion of the shooter’s shirt under UV irradiation. Figure 9d shows a photograph of the shooter’s lips under normal light for comparison. Finally, Figure 9f shows the ground located below the shot carried out with the 5.56 mm rifle. The presence of these luminescent GSR on the shooter’s clothes and hands may be associated with the high power and energy of the employed ammunition, resulting in greater residue formation and spreading. Green GSR were also observed on the firearm, such as on the trigger, magazine, and forward assist (Figure S6, in Supporting Information). Given these findings, the significant potential of the four m-LnMOFsto selectively mark (through their tunable luminescence) different types of ammunition and weapons is quite clear.

One of the challenges to be overcome when producing bullets is the noninterference of the bullet in gunpowder combustion, ensuring that the ammunition does not have any impact on its ballistics, more precisely on its power. To infer whether a given bullet interferes with the energy of rifle shots, ballistic FPS can be assessed and conveniently transformed into speed (m s^–1^) or even into the energy corresponding to the shot (in Joules). Therefore, a device called a chronograph (Figure S7, in Supporting Information) is used to determine this important ballistic factor.

**Figure 10:**
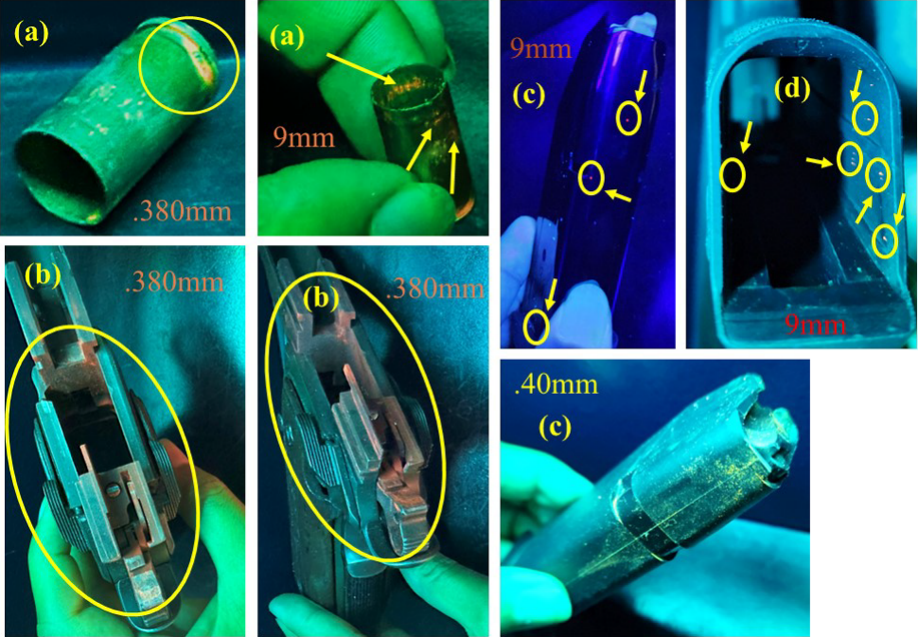
(a) GSR orange and red in aspent cartridge of 0.380 and 9 mm pistols, respectively; (b) GSR orange in the internal compartment of the 0.380 mm pistol; (c) GSR red and yellow in magazines of the 9 and 0.40 mm pistols, respectively; (d) red GSR in the magazine insertion location, in the 9 mm pistol.

The FPS values were measured before and after inserting the marker into the gunpowder, that is, with a bullet containing no marker and with a marked bullet. In the marked bullets, approximately 0.1949 g of the propellant charge was removed, and 180, 250, and 350 mg of marker were added to each bullet. This criterion was adopted as a safety measure since the propellant charge in a 5.56 mm rifle bullet is high, resulting in high-energy shots. Furthermore, the best mass quantity of tracers to add without compromising the shots can also be preliminarily estimated with three different amounts of tracers. Firing the 5.56 mm rifle with the ammunition without the tracer resulted in a projectile FPS of 2,664 ft/s. Shots fired with the ammunition marked with compound [Eu_0.007_Gd_0.3_Tb_0.693_(Hbtec)]_*n*_**1** resulted in FPS values of 2309, 2,475, and 2,552 ft/s, respectively. Surprisingly, the results reveal that the addition of 350 mg of this marker to the gunpowder has less impact on speed reduction (higher FPS) of the projectile expelled from the rifle barrel without compromising the accuracy or energy of the shot, as a reduction of just under 5% in the ballistic FPS is noted compared to the unmarked ammunition. This is significant, as it indicates that our markers do not alter the powder combustion and, consequently, shot response times. This is extremely relevant since the initial proposal is to use such markers in the ammunition of different state police forces. It is worth noting again that ammunition marker assessments for long-range weapons (such as rifles) are unprecedented, and no other study has been reported in the literature. Figure 10a also shows that the m-LnMOFs mark the fired cartridges (shown in Figure 10a for 0.380 mm GC MD Imbel pistol and for 9 mm G2C Taurus pistol).

Figure 10b shows the intense orange markings in the internal compartments of the 0.380 mm GC MD1 Imbel pistol. This is also quite relevant since these orange GSRs can remain for months inside the fired pistol, becoming an important factor for efficient material collection analysis following firing. Finally, Figure 10c shows the yellow residue markings in the magazine of the 0.40 mm pistol and red magazine residue markings of the 9 mm pistol, in addition to red GSRs in the magazine insertion site of the 9 mm pistol (Figure 10d). Among the three pistol ammunitions, the one with the highest energy is that of the 0.40 mm caliber pistol, due to its greater propellant charge. However, no greater scattering or formation pattern of these LGRS was observed with varying calibers.

Some studies have addressed the synthesis and application of LnMOFs as gunshot residue markers. In one of these studies, Lucena et al. reported a homometallic metal–organic Dy framework that was used as a GSR marker. According to its CIE diagram (obtained from Spectra Lux software),^38^ the emission of this compound is centered in yellow. However, no appreciable LGSR in the forensic environment visualization was observed.^28^ In another study, the same authors describe the synthesis of a metal–organic Eu framework where, unlike the previous compound, easily identifiable luminescent GSR were found both on the weapon surface and on the shooter’s hand.^30^

## 4 Conclusions

The possibility of obtaining multicolored emissions for m-LnMOFs is practically inexhaustible, given the number of efficient ligands that can sensitize different Ln^3+^ ions and the unlimited choice of the molar fractions of these metal ions that may be used in compound preparation. Thus, without a shadow of a doubt, these compounds assume a prominent place in Inorganic Chemistry, mainly due to their long-awaited tunable luminescence.

In this study, a new class of m-LnMOFs was prepared using the lanthanide ions Eu^3+^, Gd^3+^, and Tb^3+^ alongside 1,2,4,5-benzenetetracarboxylic acid, which proved to be an efficient Eu^3+^ and Tb^3+^ sensitizer. The investigated compounds form a class of isostructural complexes with each other and with their homometallic congeners. [Tb(Hbtec)]_n_,^29^ [Eu(Hbtec)]_*n*_^31^ Additionally, the mixed networks synthesized herein were fully characterized through different analytical and spectroscopic techniques, as well as an in-depth photophysical study. The emission spectra of the synthesized compounds exhibit the characteristic transitions of both Eu^3+^ and Tb^3+^, proving the insertion of these species into the polymeric network. The mixed compound formed only by these two metal cations [Eu_0.0167_Tb_0.9833_(Hbtec)]_*n*_**4** presented an energy transfer efficiency (η_ET_) Tb^3+^ → Eu^3+^ of about 38%, considerably lower than that of its counterpart [Eu_0.1_Tb_0.9_(Hbtec)]_*n*_^33^ previously reported by our group (η_ET_ = 98%). However, obtaining the expected tunable luminescence was only possible after inserting Gd^3+^ into the networks, as this ion plays a role in increasing the distance between the Eu^3+^ and Tb^3+^ centers, decreasing Tb^3+^ → Eu^3+^ transfer probability, and allowing light emissions closer to the green region, such as for m-LnMOF[Eu_0.014_Gd_0.8_Tb_0.186_(Hbtec)]_*n*_**2,** a yellow region emitter (*x* = 0.454 and *y* = 0.470).

Due to the high thermal stability of these polymeric networks and their tunable luminescence, these compounds are very efficient as selective firearm ammunition markers, allowing for the differentiation of the type of firearm ammunition/caliber. In addition, luminescent yellow, orange, red, and green residues were generated after firing, proving efficiency not only in selective ammunition marking but also concerning the firearms themselves and even the shooter.

This study is paramount, given the need for stricter ammunition sale controls and scientific assistance for investigative police forces. To the best of our knowledge, no studies involving tunable m-LnMOFs luminescence for use in marking or differentiating ammunition are available, nor have tests been performed with long-range, high-energy weapons, such as rifles. Therefore, in addition to a pioneering nature, this study plays a fundamental role in providing returns to taxpayers in an attempt to assist Public Security Policies.

## Supporting Information Available

Tables with ICP, infrared spectroscopy, X-ray and lifetimes data, figures of vibrational spectra, TG curves, X-ray diffractograms, lifetime curves, luminescent green particles detected in 5.56 mm Taurus T4 rifle, and forensic experimental details (PDF)

## References

[ref1] You L. X.; Ren B. Y.; He Y. K.; Wang S. J.; Sun Y. G.; Dragutan V.; Xiong G.; Ding F. Structural Features of Lanthanide Coordination Polymers with Catalytic Properties. J. Mol. Struct. 2024, 1304, 137687 10.1016/j.molstruc.2024.137687.

[ref2] Batten S.; Neil R. C.; Chen X.-M.; Garcia-Martinez J.; Kitagawa S.; Ohrstrom L.; O’Keeffe M.; Suh M. P.; Reedijk J. Terminology of Metal–Organic Frameworks Andcoordination Polymers (IUPAC Recommendations 2013). Pure Appl. Chem. 2013, 85 (8), 1715–1724. 10.1351/PAC-REC-12-11-20.

[ref3] Echenique-Errandonea E.; Mendes R. F.; Figueira F.; Choquesillo-Lazarte D.; Beobide G.; Cepeda J.; Ananias D.; Rodríguez-Diéguez A.; Almeida Paz F. A.; Seco J. M. Multifunctional Lanthanide-Based Metal-Organic Frameworks Derived from 3-Amino-4-Hydroxybenzoate: Single-Molecule Magnet Behavior, Luminescent Properties for Thermometry, and CO_2_Adsorptive Capacity. Inorg. Chem. 2022, 61 (33), 12977–12990. 10.1021/acs.inorgchem.2c00544. 35939069 PMC9406282

[ref4] Li L.; Wang J.; Zhang Z.; Yang Q.; Yang Y.; Su B.; Bao Z.; Ren Q. Inverse Adsorption Separation of CO_2_ /C_2_H_2_ Mixture in Cyclodextrin-Based Metal-Organic Frameworks. ACS Appl. Mater. Interfaces 2019, 11 (2), 2543–2550. 10.1021/acsami.8b19590. 30565914

[ref5] Wang Y.; Yan J.; Wen N.; Xiong H.; Cai S.; He Q.; Hu Y.; Peng D.; Liu Z.; Liu Y. Metal-Organic Frameworks for Stimuli-Responsive Drug Delivery. Biomaterials 2020, 230, 119619 10.1016/j.biomaterials.2019.119619. 31757529

[ref6] Hao M.; Qiu M.; Yang H.; Hu B.; Wang X. Recent Advances on Preparation and Environmental Applications of MOF-Derived Carbons in Catalysis. Sci. Total Environ. 2021, 760, 143333 10.1016/j.scitotenv.2020.143333. 33190884

[ref7] Wang Y.; Liu Y.; Wang H.; Liu W.; Li Y.; Zhang J.; Hou H.; Yang J. Ultrathin NiCo-MOF Nanosheets for High-Performance Supercapacitor Electrodes. ACS Appl. Energy Mater. 2019, 2 (3), 2063–2071. 10.1021/acsaem.8b02128.

[ref8] Dang W.; Sun Y.; Jiao H.; Xu L.; Lin M. AuNPs-NH2/Cu-MOF Modified Glassy Carbon Electrode as Enzyme-Free Electrochemical Sensor Detecting H_2_O_2_. J. Electroanal. Chem. 2020, 856, 113592 10.1016/j.jelechem.2019.113592.

[ref9] Tian H.; Zhang M.; Jin G.; Jiang Y.; Luan Y. Cu-MOF Chemodynamic Nanoplatform via Modulating Glutathione and H_2_O_2_ in Tumor Microenvironment for Amplified Cancer Therapy. J. Colloid Interface Sci. 2021, 587, 358–366. 10.1016/j.jcis.2020.12.028. 33360905

[ref10] Liu H.; Lu J.; Liu Z.; Wang S.; Yan H.; Tian H. Proton Conducting Behavior of a Microporous Metal-Organic Framework Assisted by Ligand Isomerization. J. Solid State Chem. 2020, 290, 121570 10.1016/j.jssc.2020.121570.

[ref11] Cepeda J.; Pérez-Yáñez S.; Beobide G.; Castillo O.; García J. Á.; Luque A. Photoluminescence Modulation in Lanthanide(III)/Pyrazine-2,5-Dicarboxylato/Nitrato Frameworks. Eur. J. Inorg. Chem. 2015, 2015 (26), 4318–4328. 10.1002/ejic.201500484.

[ref12] Psalti A. E.; Andriotou D.; Diamantis S. A.; Chatz-Giachia A.; Pournara A.; Manos M. J.; Hatzidimitriou A.; Lazarides T. Mixed-Metal and Mixed-Ligand Lanthanide Metal-Organic Frameworks Based on 2,6-Naphthalenedicarboxylate: Thermally Activated Sensitization and White-Light Emission. Inorg. Chem. 2022, 61 (30), 11959–11972. 10.1021/acs.inorgchem.2c01703. 35861587

[ref13] Igoa F.; Peinado G.; Suescun L.; Kremer C.; Torres J. Design of a White-Light Emitting Material Based on a Mixed-Lanthanide Metal Organic Framework. J. Solid State Chem. 2019, 279 (June), 120925 10.1016/j.jssc.2019.120925.

[ref14] Sun D.; Ye L.; Sun F.; García H.; Li Z. From Mixed-Metal MOFs to Carbon-Coated Core-Shell Metal Alloy@Metal Oxide Solid Solutions: Transformation of Co/Ni-MOF-74 to Co_x_Ni_1-X_@CoyNi1-YO@C for the Oxygen Evolution Reaction. Inorg. Chem. 2017, 56 (9), 5203–5209. 10.1021/acs.inorgchem.7b00333. 28387519

[ref15] Yin H. Q.; Yin X. B. Metal-Organic Frameworks with Multiple Luminescence Emissions: Designs and Applications. Acc. Chem. Res. 2020, 53 (2), 485–495. 10.1021/acs.accounts.9b00575. 31999097

[ref16] Wang J.-X.; Yin J.; Shekhah O.; Bakr O. M.; Eddaoudi M.; Mohammed O. F. Energy Transfer in Metal–Organic Frameworks for Fluorescence Sensing. ACS Appl. Mater. Interfaces 2022, 14 (8), 9970–9986. 10.1021/acsami.1c24759. 35175725 PMC8895374

[ref17] Abednatanzi S.; Gohari Derakhshandeh P.; Depauw H.; Coudert F. X.; Vrielinck H.; Van Der Voort P.; Leus K. Mixed-Metal Metal-Organic Frameworks. Chem. Soc. Rev. 2019, 48, 2535–2565. 10.1039/C8CS00337H. 30989162

[ref18] Wei Z.; Ren L.; Xiao X.; Zhang Q.; Huang J.; Liu R.; Zhao S.; Xu W. Biomimetic Mineralization of Nanoscale Lanthanide Metal-Organic Frameworks with Thermo-Sensitive Polymer as Organic Ligands for Solvent Recognition and Water Detection. J. Solid State Chem. 2019, 277 (July), 594–601. 10.1016/j.jssc.2019.07.010.

[ref19] Gao Y.; Yu G.; Liu K.; Wang B. Luminescent Mixed-Crystal Ln-MOF Thin Film for the Recognition and Detection of Pharmaceuticals. Sens Actuators B Chem. 2018, 257, 931–935. 10.1016/j.snb.2017.10.180.

[ref20] Zhang F.; Li J.; Zhao Z.; Wang F.; Pu Y.; Cheng H. Mixed-LnMOFs with Tunable Color and White Light Emission Together with Multi-Functional Fluorescence Detection. J. Solid State Chem. 2019, 280, 120972 10.1016/j.jssc.2019.120972.

[ref21] Qiu Y. C.; Yuan S.; Li X. X.; Du D. Y.; Wang C.; Qin J. S.; Drake H. F.; Lan Y. Q.; Jiang L.; Zhou H. C. Face-Sharing Archimedean Solids Stacking for the Construction of Mixed-Ligand Metal-Organic Frameworks. J. Am. Chem. Soc. 2019, 141 (35), 13841–13848. 10.1021/jacs.9b05580. 31343873

[ref22] Blakey L. S.; Sharples G. P.; Chana K.; Birkett J. W. Fate and Behavior of Gunshot Residue—A Review. J. Forensic Sci. 2018, 63 (1), 9–19. 10.1111/1556-4029.13555. 28543548

[ref23] Wallace J. S. Chemical Analysis of Firearms, Ammunition, and Gunshot Residue, 2nd ed.; CRC Press, 2018.

[ref24] Arouca A. M.; Lucena M. A. M.; Rossiter R. J.; Talhavini M.; Weber I. T. Use of Luminescent Gunshot Residues Markers in Forensic Context—Part II. Forensic Sci. Int. 2017, 281, 161–170. 10.1016/j.forsciint.2017.09.022. 29156218

[ref25] Lucena M. A. M.; Arouca A. M.; Talhavini M.; Alves-Júnior S.; Weber I. T. Ammunition Encoding by Means of Co-Doped Luminescent Markers. Microchem. J. 2019, 145, 539–546. 10.1016/j.microc.2018.09.013.

[ref26] Weber I. T.; De Melo A. J. G.; Lucena M. A. D. M.; Rodrigues M. O.; Alves Junior S. High Photoluminescent Metal - Organic Frameworks as Optical Markers for the Identification of Gunshot Residues. Anal. Chem. 2011, 83 (12), 4720–4723. 10.1021/ac200680a. 21585195

[ref27] Lucena M. A. M.; Câmara S. S.; Talhavini M.; Weber I. T. Yttrium Orthovanadates Phosphors as Up-Conversion Luminescent Markers for Gunshot Residue Identification. J. Lumin. 2022, 250, 119020 10.1016/j.jlumin.2022.119020.

[ref28] Melo Lucena M. A.; Rodrigues M. O.; Gatto C. C.; Talhavini M.; Maldaner A. O.; Alves S.; Weber I. T. Synthesis of [Dy(DPA)(HDPA)] and Its Potential as Gunshot Residue Marker. J. Lumin. 2016, 170, 697–700. 10.1016/j.jlumin.2015.04.010.

[ref29] Silva M. A.; de Campos N. R.; Ferreira L. A.; Flores L. S.; Júnior J. C. A.; dos Santos G. L.; Corrêa C. C.; dos Santos T. C.; Ronconi C. M.; Colaço M. V.; Simões T. R. G.; Marques L. F.; Marinho M. V. A New Photoluminescent Terbium(III) Coordination Network Constructed from 1,2,4,5-Benzenetetracarboxylic Acid: Synthesis, Structural Characterization and Application as a Potential Marker for Gunshot Residues. Inorg. Chim. Acta 2019, 495 (May), 118967 10.1016/j.ica.2019.118967.

[ref30] Lucena M. A. M.; Oliveira M. F. L.; Arouca A. M.; Talhavini M.; Ferreira E. A.; Alves S.; Veiga-Souza F. H.; Weber I. T. Application of the Metal-Organic Framework [Eu(BTC)] as a Luminescent Marker for Gunshot Residues: A Synthesis, Characterization, and Toxicity Study. ACS Appl. Mater. Interfaces 2017, 9 (5), 4684–4691. 10.1021/acsami.6b13474. 27936564

[ref31] Marques L. F.; Júnior J. C. A.; Dos Santos G. L.; Colaço M. V.; Barroso R. C.; Ferreira F. F.; Dos Santos M. V.; De Campos N. R.; Marinho M. V.; Jesus L. T.; Freire R. O. New Eu^III^ Pyromellitic Metal-Organic Framework of Intense Red-Orange Luminescence and High Thermal Stability for Marking in Gunshot Residues. J. Phys. Chem. C 2020, 124 (18), 9996–10006. 10.1021/acs.jpcc.0c01374.

[ref32] Lucena M. A. M.; Ordoñez C.; Weber I. T.; Torre M.; García-Ruiz C.; López-López M. Investigation of the Use of Luminescent Markers as Gunshot Residue Indicators. Forensic Sci. Int. 2017, 280, 95–102. 10.1016/j.forsciint.2017.09.013. 28985595

[ref33] De Oliveira Silva J. P.; Colaço M. V.; De Souza L. A.; Dos Santos M. V.; Pugina R. S.; Marques L. F. Exploring the Intense Lanthanide Luminescence and High Thermal Stability in a New Mixed Eu^3+^/Tb^3+^Organic Framework Series for Marking in Gunshot Residues. J. Phys. Chem. C 2022, 126 (38), 16568–16577. 10.1021/acs.jpcc.2c05594.

[ref34] Deacon G. B.; Phillips R. J. Relationships between the Carbon-Oxygen Stretching Frequencies of Carboxylato Complexes and the Type of Carboxylate Coordination. Coord. Chem. Rev. 1980, 33, 227–250. 10.1016/S0010-8545(00)80455-5.

[ref35] Guo L.; Wu G.; Li H. H. Synthesis, Crystal Structures, Thermal and Luminescent Properties of Rare Earth Metal Complexes with 1,2,4,5-Benzenetetracarboxylic Acid. J. Chem. Crystallogr. 2012, 42 (3), 192–198. 10.1007/s10870-011-0223-3.

[ref36] Tymiński A.; Grzyb T. Are Rare Earth Phosphates Suitable as Hosts for Upconversion Luminescence. Studies on Nanocrystalline REPO_4_ (RE = Y, La, Gd, Lu) Doped with Yb^3+^ and Eu^3+^, Tb^3+^, Ho^3+^, Er^3+^ or Tm^3+^ Ions. J. Lumin. 2017, 181, 411–420. 10.1016/j.jlumin.2016.09.028.

[ref37] Jia Y. Q. Crystal Radii and Effective Ionic Radii of the Rare Earth Ions. J. Solid StateChem. 1991, 95, 184 10.1016/0022-4596(91)90388-X.

[ref38] Santa-Cruz P. A.; Teles F. S. Spectra Lux Softwaere v.2.0, Ponto Quântico Nanodispositivos, UFPE. https://sites.ufpe.br/vitrine/softwares/spectra-lux/.

[ref39] SeethaLekshmi S.; Ramya A. R.; Reddy M. L. P.; Varughese S. Lanthanide Complex-Derived White-Light Emitting Solids: A Survey on Design Strategies. Journal of Photochemistry and Photobiology C: Photochemistry Reviews 2017, 33, 109–131. 10.1016/j.jphotochemrev.2017.11.001.

[ref40] Ma L. N.; Liu Y.; Li Y. Z.; Hu Q. X.; Hou L.; Wang Y. Y. Three Lanthanide Metal-Organic Frameworks Based on an Ether-Decorated Polycarboxylic Acid Linker: Luminescence Modulation, CO_2_ Capture and Conversion Properties. Chem. Asian J. 2020, 15 (1), 191–197. 10.1002/asia.201901506. 31782903

[ref41] Wu J.; Zhang H.; Du S. Tunable Luminescence and White Light Emission of Mixed Lanthanide-Organic Frameworks Based on Polycarboxylate Ligands. J. Mater. Chem. C Mater. 2016, 4 (16), 3364–3374. 10.1039/C5TC04432D.

