# Supplementary material for: Use of Luminescence Modulation in a New Series of Mixed Lanthanide Metal–Organic Frameworks for Selective Firearm Ammunition Marking
Source: ACS Omega. 2024 Dec 12;9(51):50579–88. doi: 10.1021/acsomega.4c08401 (PMC11683623; doi:10.1021/acsomega.4c08401)
Supplement: Supplementary file 1 — ao4c08401_si_001.pdf [file ao4c08401_si_001.pdf]

Use of luminescence modulation in a new series of mixed lanthanide metal-organic frameworks for selective firearm ammunition marking

Júlia P. De Oliveira Silva [a], Marcos V. Colaço [b], Alexandre R. Camara [c], Renato de Almeida Pereira [a], Eduardo de Oliveira Fernandes [a], Claudiane C. Canuto [a] , Diego R. Carvalhosa [a] and Lippy F. Marques [a] *

[a] Grupo de Química de Coordenação e Espectroscopia de Lantanídeos (GQCEL), Instituto de Química, Universidade do Estado do Rio de Janeiro, Rio de Janeiro-RJ, 20550-013, Brazil

[b] Laboratório de Física Médica (LabFisMed), Instituto de Física, Universidade do Estado do Rio de Janeiro, Rio de Janeiro-RJ, 25550-013, Brazil

[c] Departamento de Eletrônica Quântica, Instituto de Física, Universidade do Estado do Rio de Janeiro, Rio de Janeiro-RJ, 25550-013, Brazil

* Email: lippymarquesuerj@gmail.com

Table S1. Compounds formula and ICP data for all m-LnMOFs.

m-LnMOF	Eu: Gd: Tb (ICP analysis)
$[Eu_{0.014}Gd_{0.8}Tb_{0.186}(Hbtec)]_n$	0.0153: 0.8008: 0.1839
$[Eu_{0.014}Gd_{0.9}Tb_{0.086}(Hbtec)]_n$	0.0136: 0.8991: 0.0873
$[Eu_{0.0167}Tb_{0.9833}(Hbtec)]_n$	0.0170: 0.0000: 0.9830

Table S2. Main absorption bands of the ligand and synthesized mixed lanthanide metal-organic frameworks.

Compound	Wavenumber (cm^{-1})						
	ν(CO)	ν(OH)	$ν(CH)_{ArH}$	$ν(C=C)_{ArH}$	$ν_{asym}(CO_2^-)$	$ν_{sym}(CO_2^-)_1$	$ν_{sym}(CO_2^-)_2$
H_4btec	1695	3003	3055	1576/1505	-_	-_	-_
$[Tb(Hbtec)]_n$*	1669	2762	3052	1596/1574	1612	1461	1373
$[Eu(Hbtec)]_n$*	1671	2772	3050	1597/1505	1613	1460	1373
$[Eu_{0.007}Gd_{0.3}Tb_{0.693}(Hbtec)]_n$ **1**	1673	2771	3056	1566/1530/ 1490	1613	1463	1373
$[Eu_{0.014}Gd_{0.8}Tb_{0.186}(Hbtec)]_n$ **2**	1669	2772	3056	1564/1531/ 1490	1613	1461	1373
$[Eu_{0.014}Gd_{0.9}Tb_{0.086}(Hbtec)]_n$ **3**	1671	2771	3050	1565/1530/ 1490	1613	1461	1372
$[Eu_{0.0167}Tb_{0.9833}(Hbtec)]_n$ **4**	1672	2775	3051	1567/1531/ 1490	1614	1462	1380

*Legend: *The spectroscopy datas for $[Tb(Hbtec)]_n$ and $[Eu(Hbtec)]_n$ were previously reported by[19].*

Table S3. Primitive unit cell volumes for **1-4** mixed lanthanide metal-organic frameworks, and their quality crystallographic parameters.

Compound		Volume (Å^3)	R_{wp} (%)	R_{exp} (%)	R_{Bragg} (%)	GOF
$[Eu_{0.007}Gd_{0.3}Tb_{0.693}(Hbtec)]_n$	**1**	457.14(9)	4.146	2.807	0.199	1.476
$[Eu_{0.014}Gd_{0.8}Tb_{0.186}(Hbtec)]_n$	**2**	459.37(2)	5.390	3.435	0.407	1.569
$[Eu_{0.014}Gd_{0.9}Tb_{0.086}(Hbtec)]_n$	**3**	460.57(3)	5.331	3.513	0.275	1.517
$[Eu_{0.0167}Tb_{0.9833}(Hbtec)]_n$	**4**	457.64(2)	6.074	3.001	0.439	2.023
$[Tb(Hbtec)]_n^*$		456.51(1)	3.283	0.974	0.210	3.368
$[Eu(Hbtec)]_n^*$		461.63(1)	4.225	0.957	0.600	4.412

*Legend: *The DRX datas for $[Tb(Hbtec)]_n$ and $[Eu(Hbtec)]_n$ were previously reported by*[19]

Table S4. Lifetime decays for all synthesized m-LnMOFs.

Compound	t(Eu^{3+})/ms	t(Tb^{3+})/ms
$[Eu_{0.007}Gd_{0.3}Tb_{0.693}(Hbtec)]_n$ **1**	$\tau(^5D_0) = 2.643 \pm 0.002$	$\tau(^5D_4) = 1.991 \pm 0.002$
$[Eu_{0.014}Gd_{0.8}Tb_{0.186}(Hbtec)]_n$ **2**	$\tau(^5D_0) = 1.991 \pm 0.002$	$\tau(^5D_4) = 1.669 \pm 0.003$
$[Eu_{0.014}Gd_{0.9}Tb_{0.086}(Hbtec)]_n$ **3**	$\tau(^5D_0) = 1.681 \pm 0.002$	$\tau(^5D_4) = 1.769 \pm 0.009$
$[Eu_{0.0167}Tb_{0.9833}(Hbtec)]_n$ **4**	$\tau(^5D_0) = 1.614 \pm 0.004$	$\tau(^5D_4) = 0.714 \pm 0.002$

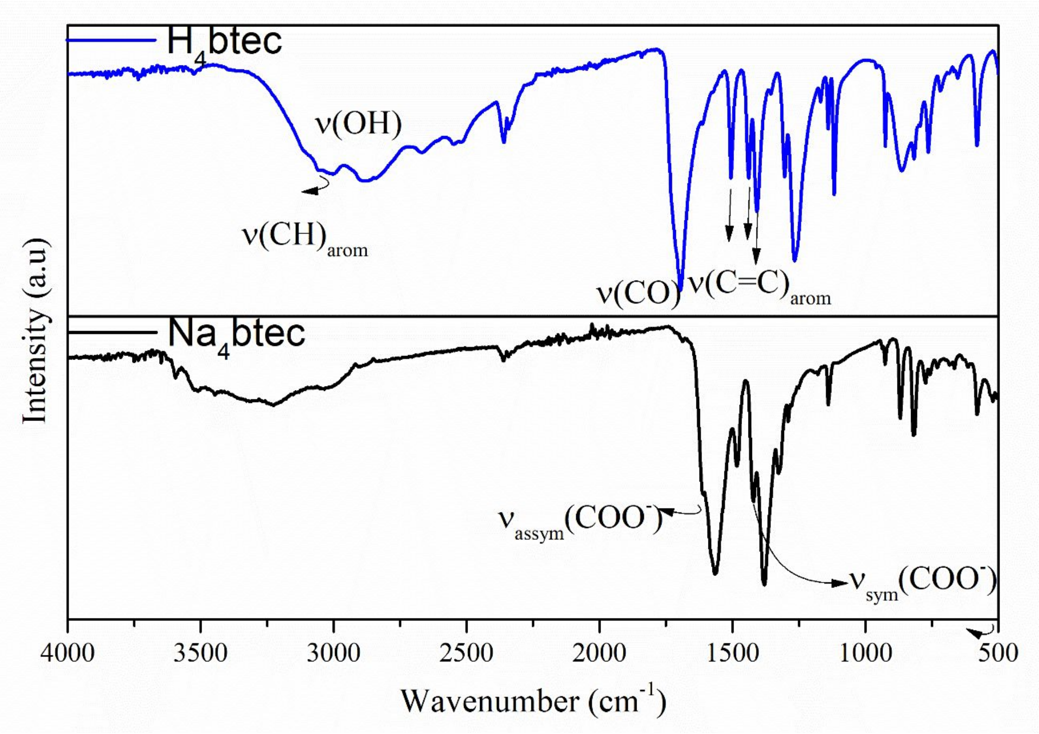

Figure S1. Vibrational Spectra for the four 1,2,4,5-benzenetetracarboxylic acid and sodium salt Na_4btec.

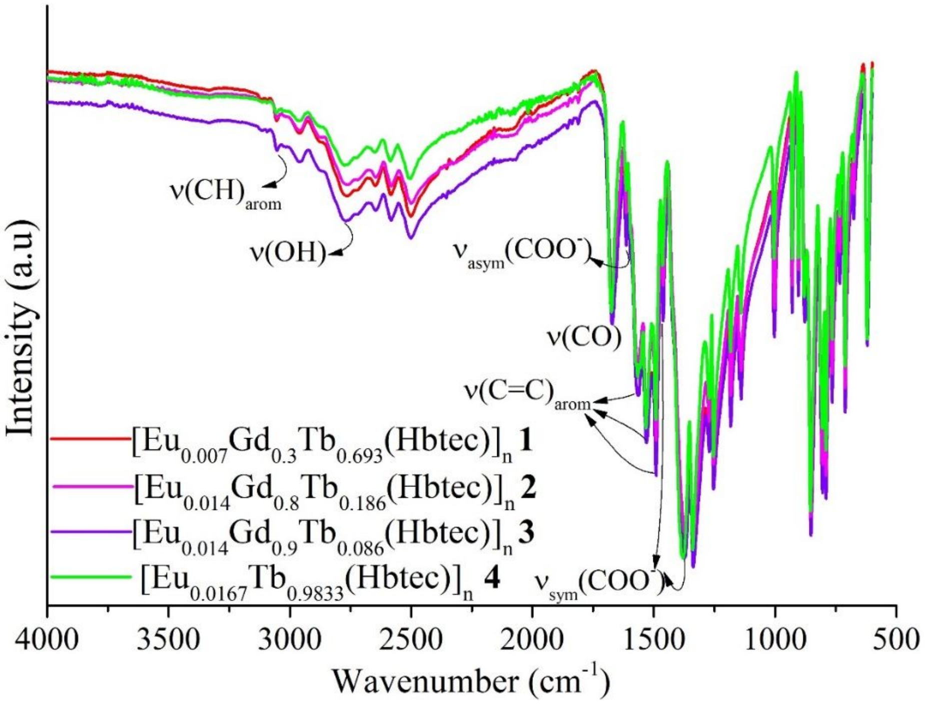

Figure S2. Vibrational Spectra for the four mixed lanthanide metal-organic frameworks.

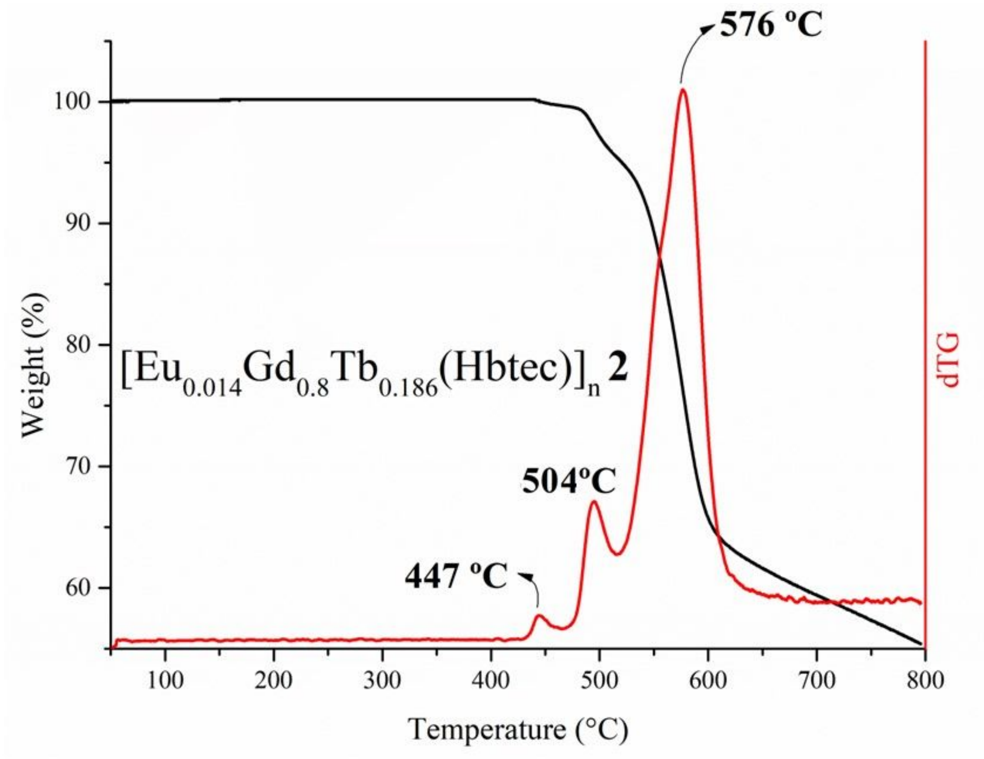
576 ºC
504ºC
447 ºC
[Eu0.014Gd0.8Tb0.186(Hbtec)]n 2
Weight (%)
dTG
Temperature (°C)
100
90
80
70
60
100 200 300 400 500 600 700 800

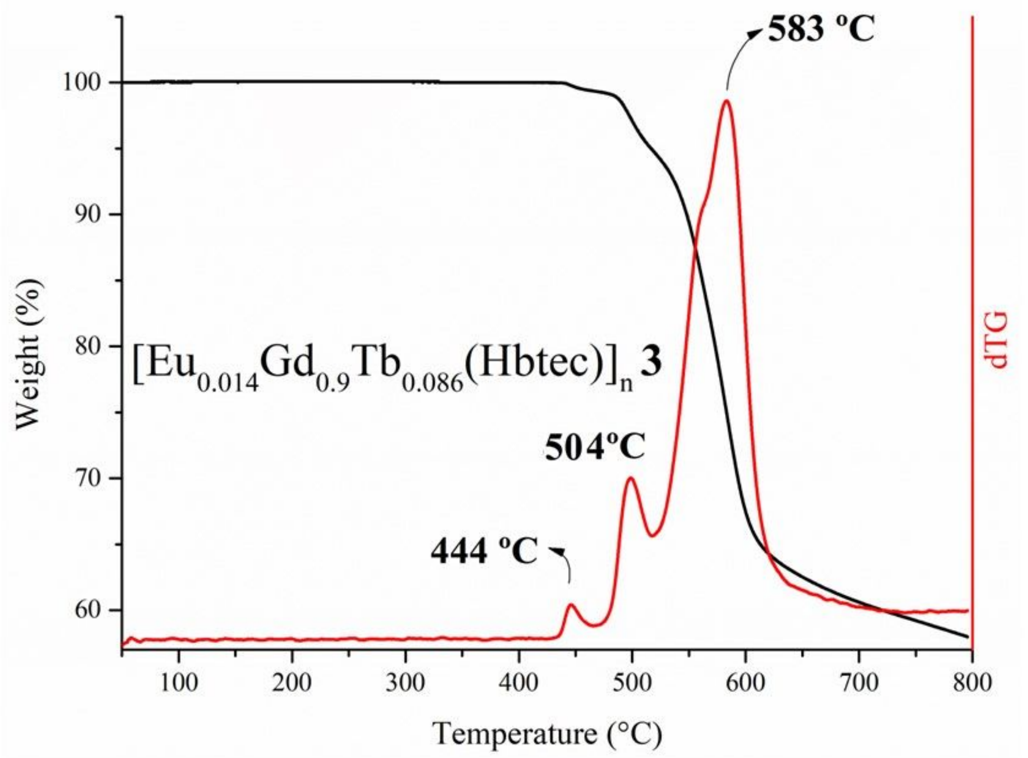
583 ºC
504ºC
444 ºC
[Eu0.014Gd0.9Tb0.086(Hbtec)]n 3
Weight (%)
dTG
Temperature (°C)
100
90
80
70
60
100 200 300 400 500 600 700 800

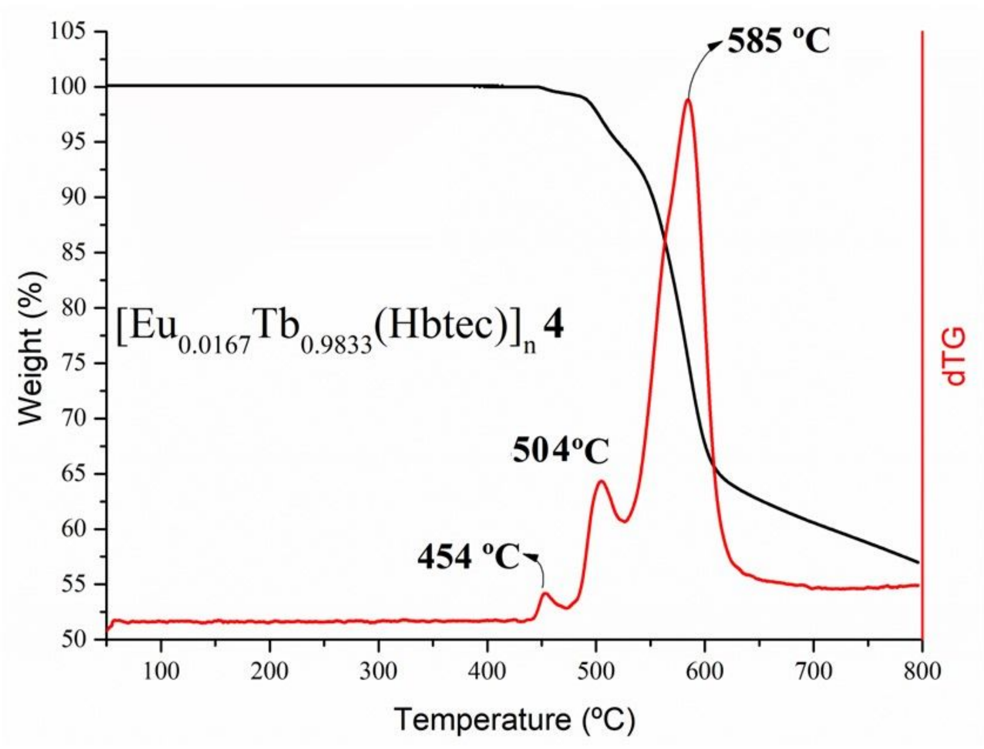

Figure S3. TG (in black) and dTG (in red) curves for the **1-3** mixed lanthanide metal-organic frameworks.

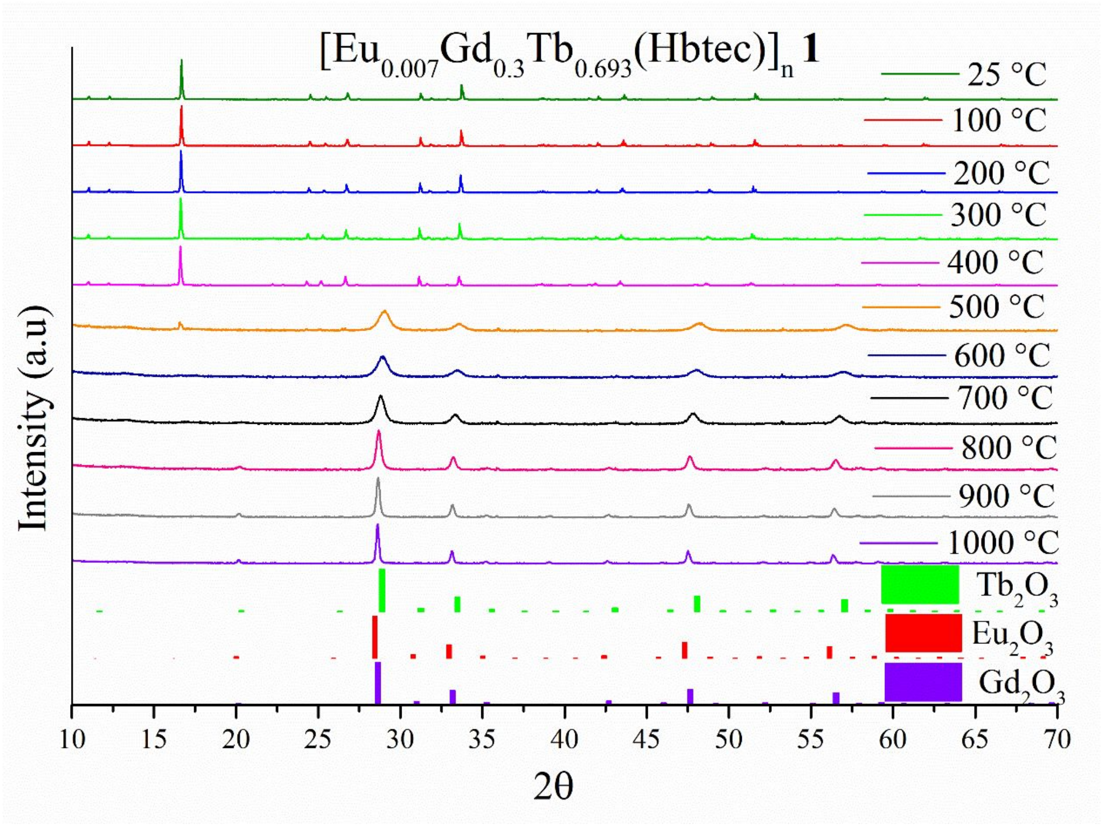

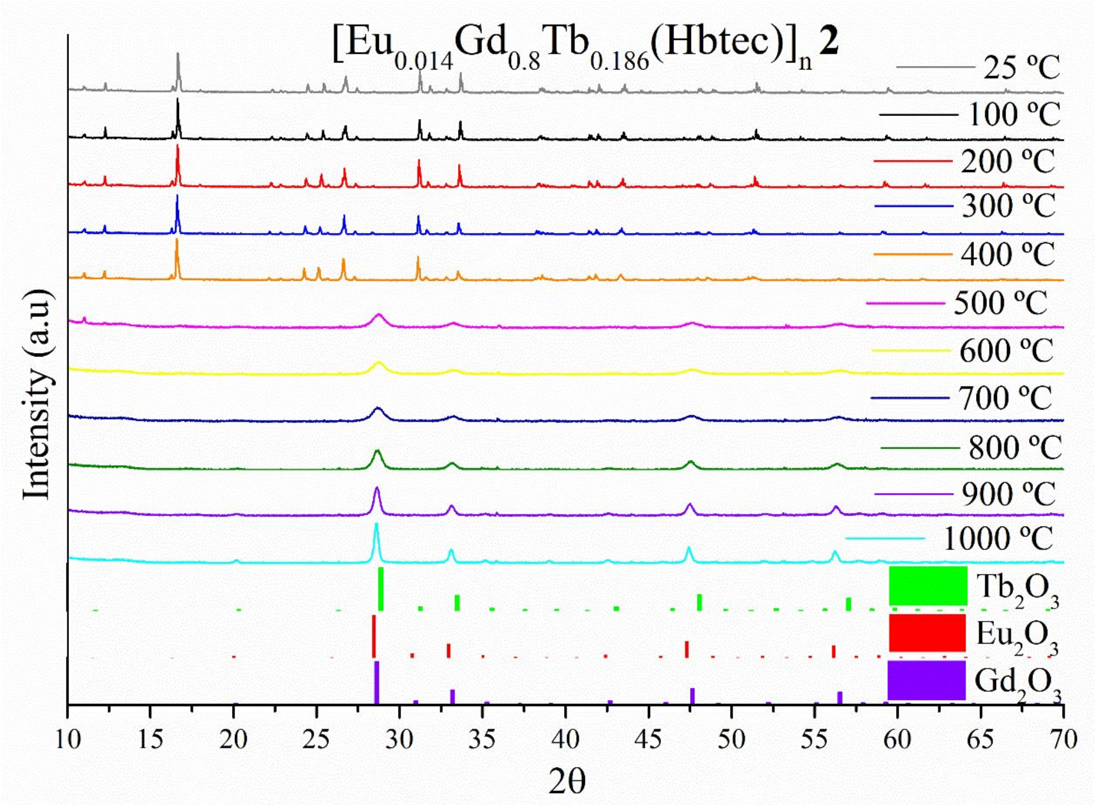
[Eu0.014Gd0.8Tb0.186(Hbtec)]n 2
25 ºC
100 ºC
200 ºC
300 ºC
400 ºC
500 ºC
600 ºC
700 ºC
800 ºC
900 ºC
1000 ºC
Tb2O3
Eu2O3
Gd2O3
Intensity (a.u)
10 15 20 25 30 35 40 45 50 55 60 65 70
2θ

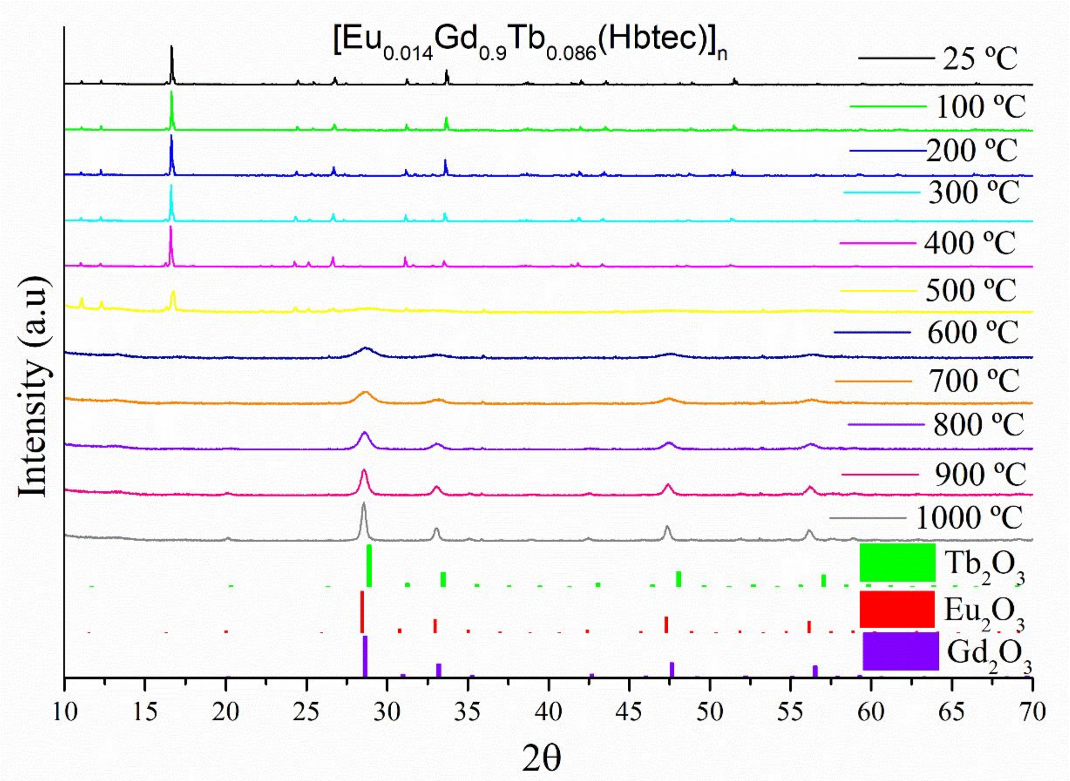
[Eu0.014Gd0.9Tb0.086(Hbtec)]n
25 ºC
100 ºC
200 ºC
300 ºC
400 ºC
500 ºC
600 ºC
700 ºC
800 ºC
900 ºC
1000 ºC
Tb2O3
Eu2O3
Gd2O3
Intensity (a.u)
10 15 20 25 30 35 40 45 50 55 60 65 70
2θ

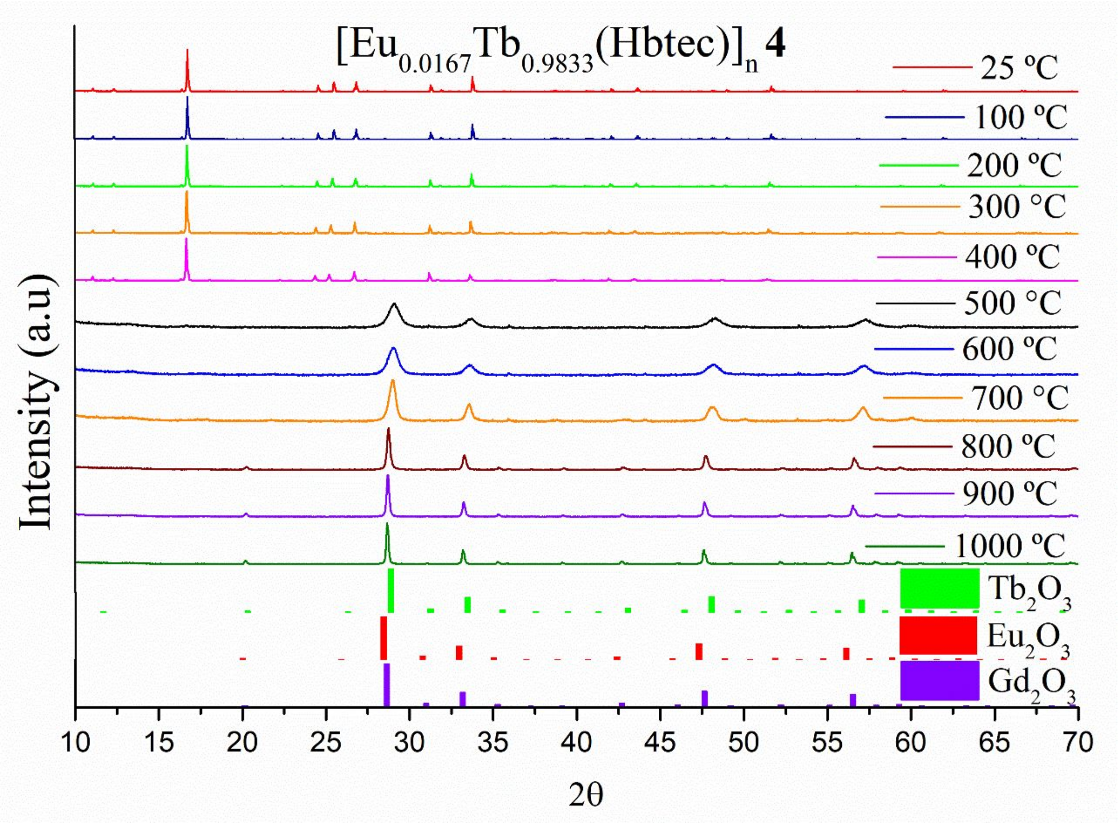

Figure S4. Diffractograms under temperature variation (25 – 1000 ºC) for the four mixed lanthanide metal-organic frameworks.

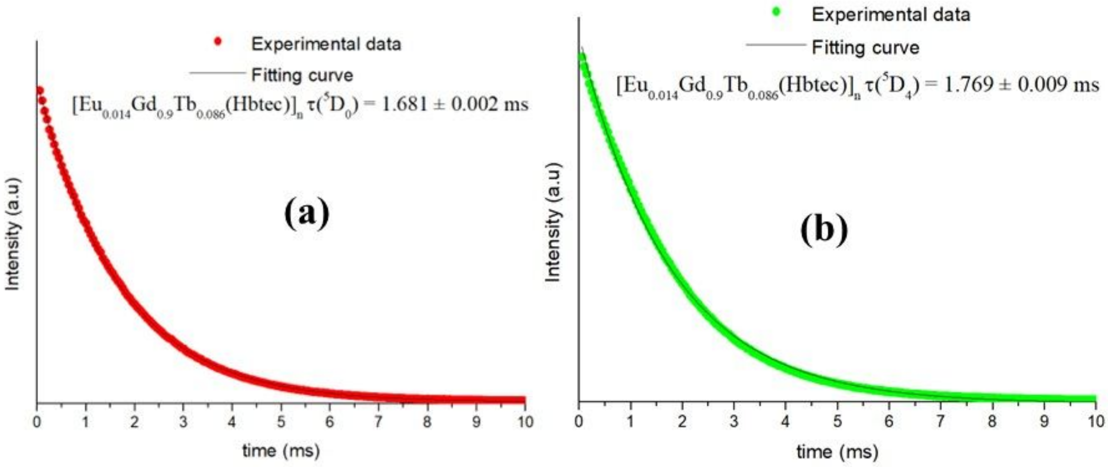

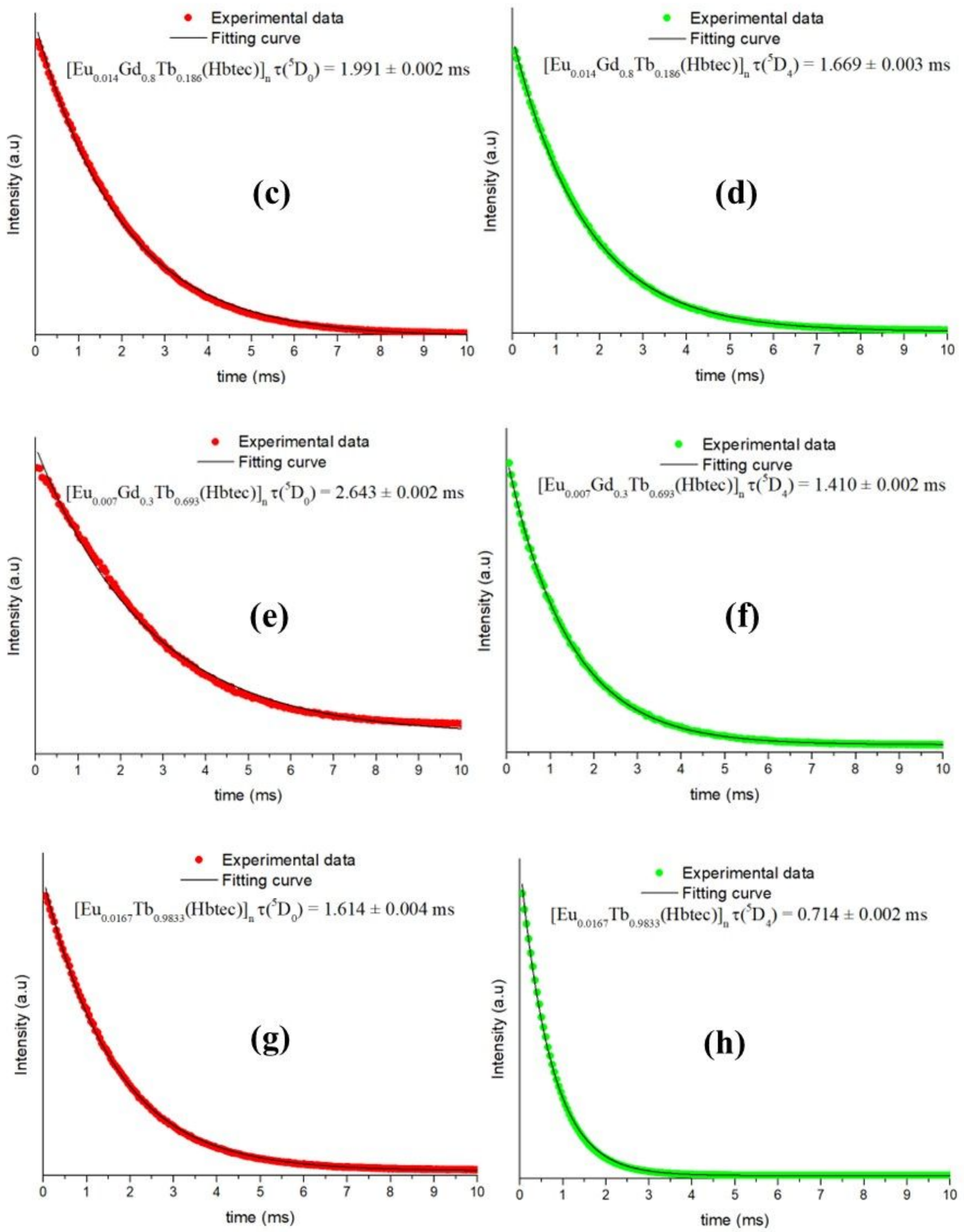

Figure S5. Lifetime decays curves for $[Eu_{0.007}Gd_{0.3}Tb_{0.693}(Hbtec)]_n$ **1** (a) and (b), $[Eu_{0.014}Gd_{0.8}Tb_{0.186}(Hbtec)]_n$ **2** (c) and (d), $[Eu_{0.014}Gd_{0.9}Tb_{0.086}(Hbtec)]_n$ **3** (e) and (f), $[Eu_{0.0167}Tb_{0.9833}(Hbtec)]_n$ **4** (g) and (h).

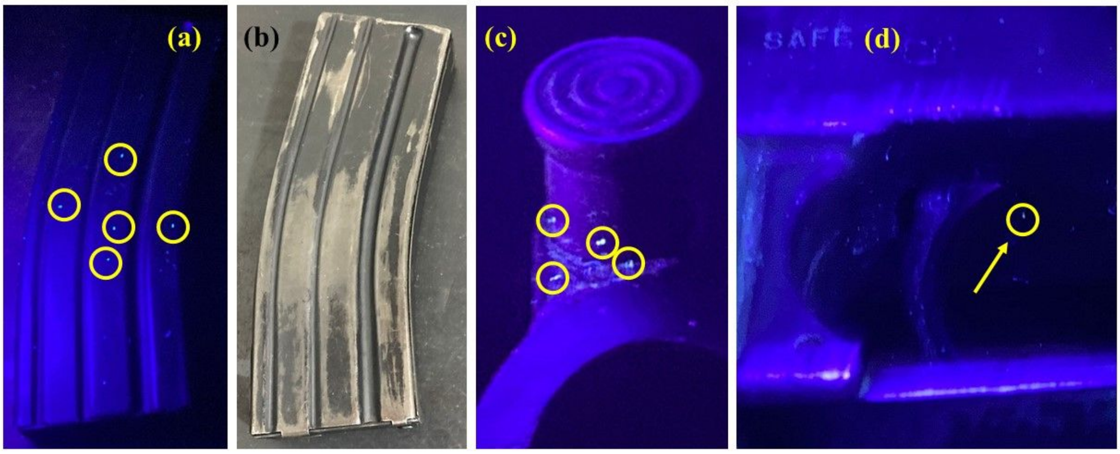

Figure S6. Luminescent green particles detected in 5.56mm Taurus T4 rifle under λ=254 nm, (a) magazine; (b) magazine under normal light; (c) forward assist and (d) trigger.

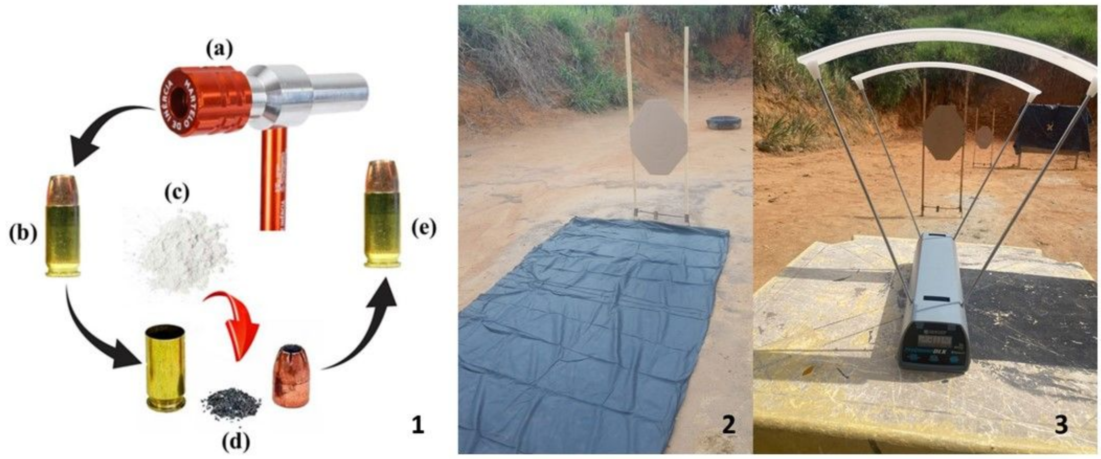

Figure S7. **1**- Preparation diagram for disassembly (with an inertial material) and addition of markers to the ammunition: (a) inertia hammer; (b) ammunition; (c) marker; (d) disassembly of ammunition for marker insertion and (e) ammunition reassembled with the marker. **2**- Shooting location, with the floor covered with black TNT tissue and a pre-assembled cardboard target. **3**- Chronograph equipment, aligned with the target. (The bullet from each ammunition passes under the white rods, and the ballistic FPS is recorded).
